# Cross-regulation of iron and phosphate homeostasis by Fep1 and Pho7 in fission yeast

**DOI:** 10.1128/spectrum.00716-26

**Published:** 2026-10-02

**Authors:** Seon-Ju Park, Yong-Joon Cho, Kyoung-Dong Kim

**Affiliations:** 1 Department of Systems Biotechnology, Chung-Ang University 65479, Anseong, Republic of Korea; 2 Department of Molecular Bioscience, Kangwon National University 34962, Chuncheon, Republic of Korea; Brown University, Providence, Rhode Island, USA

**Keywords:** iron homeostasis, phosphate homeostasis, transcriptional cross-regulation, fission yeast, Fep1, Pho7

## Abstract

Iron and phosphate are essential micronutrients with partially interconnected homeostatic control; however, the underlying transcriptional mechanisms that integrate these signals in the fission yeast *Schizosaccharomyces pombe* remain unclear. Fep1 (GATA-type repressor) is a key regulator of iron-uptake genes, whereas Pho7 (Zn_2_Cys_6_-family transcription factor) drives phosphate starvation response. However, whether each of the two factors influences the target regulon controlled by the other remains unclear. We combined genome-wide chromatin immunoprecipitation sequencing, RNA sequencing, and auxin-inducible degron-mediated acute depletion to define the joint Fep1–Pho7 transcriptional control and regulatory landscapes under defined iron and phosphate conditions. Unexpectedly, these analyses showed extensive overlap in chromatin occupancy between Fep1 and Pho7 at a subset of target promoters. Fep1 bound not only to canonical iron-uptake genes but also to the *pho7+* locus and multiple phosphate-responsive promoters, whereas Pho7 showed association with a defined subset of Fep1 targets. Acute depletion experiments showed that Fep1 loss substantially increased Pho7 chromatin occupancy at iron-responsive promoters. Under iron-depleted conditions, Pho7 contributed to the transcriptional activation of specific Fep1 target genes (*fio1+/fip1*+ and *str3+*), which indicated that Pho7 supports iron uptake gene expression when Fep1 repression is attenuated. Condition-dependent binding analyses showed that iron availability influences the relative regulatory contribution of the two factors at shared loci. These findings support a model in which Fep1 and Pho7 form a cross-regulatory module that converges on shared genes to enable iron-dependent redistribution of their influence. Together, these findings suggest a mechanistic framework for integrating iron and phosphate signals at the promoter level in fission yeast.

**IMPORTANCE:**

Iron and phosphate are essential micronutrients requiring balanced cellular levels for metabolism, redox control, and stress resistance. However, the transcriptional logic that integrates these signals remains unclear. Although fission yeast provides a powerful model for nutrient signaling, the iron-responsive factor Fep1 and the phosphate-responsive factor Pho7 have been treated as independent regulators. This study shows that Fep1 and Pho7 occupy promoters regulated by each other, supporting iron-dependent transcriptional cross-regulation. These findings suggest functional interplay between canonical iron- and phosphate-responsive pathways, although indirect metabolic coupling cannot be excluded. The iron-dependent shift in regulatory hierarchy between Fep1 and Pho7 at co-occupied promoters provides a mechanistic framework for understanding how cells can prioritize and integrate competing micronutrient demands. Therefore, cross-regulatory interactions between nutrient-specific transcription factors could indicate a general strategy for organizing multi-element homeostasis in fungi and may explain adaptation of pathogenic or commensal yeasts to fluctuating metal and phosphate environmental conditions.

## INTRODUCTION

Iron and phosphate are essential micronutrients that control cellular metabolism, genome stability, and environmental stress adaptation in eukaryotes (1). Iron functions as a cofactor for enzymes involved in respiration, DNA replication, Fe-S cluster biogenesis, and antioxidant defense; hence, its availability is critical for cell survival (2–5). Phosphate is a core component of ATP, nucleic acids, phospholipids, and signaling molecules, and its scarcity triggers genome-wide transcriptional reprogramming in response to phosphate starvation (6). Although iron and phosphate are regulated through distinct biosynthetic and signaling pathways, emerging evidence suggests that these nutrient-sensing mechanisms are integrated to coordinate cellular responses to multiple nutrient limitations (1, 2, 7–9).

An intricate cross talk has been identified between iron and phosphate homeostasis in various organisms, whereby changes in the availability of one micronutrient influence the regulation of the other. In *Saccharomyces cerevisiae*, the transcription factor Aft2 coordinates iron metabolism with phosphate acquisition and provides a molecular link between the two nutrient-sensing pathways (10). Similar metabolic integration has been observed in bacteria and plants, where iron–phosphate cross talk modulates virulence, nutrient utilization efficiency, and stress tolerance (11–17). *Schizosaccharomyces pombe* is considered the leading model organism for nutrient signaling and chromatin regulation; however, the molecular mechanisms underlying its iron–phosphate cross talk remain largely uninvestigated.

In *S. pombe*, iron homeostasis is controlled by Fep1, which is a GATA-type transcriptional repressor that binds to the canonical motif 5′-(A/T)GATA(A/T)-3′ in the promoters of iron uptake genes to repress their expression under iron-replete conditions (18). Fep1 functions as a central node in the iron-responsive transcriptional network. Its activity is dynamically regulated by Fe-S cluster binding and interaction with the glutaredoxin Grx4, which acts as an iron sensor that couples iron levels to Fep1-mediated transcriptional repression (3, 19). Under iron-depleted conditions, Grx4–Fra2-dependent signaling triggers dissociation of Fep1 from target promoters and promotes the assembly of the Php2/3/5-Php4 CCAAT-binding complex, which remodels iron-responsive gene expression to prioritize iron-saving pathways. The key target genes that are repressed by Fep1 under iron-replete conditions include those encoding components of the reductive iron uptake pathway (*fio1+* and *fip1+*), siderophore-iron uptake system (*str1+* and *str2+*), heme transporter (*str3+*), and other iron acquisition factors (20–25). Thus, the Fep1-mediated iron regulation is vital, as highlighted by the severe phenotypes observed upon its perturbation, which disrupt intracellular iron balance and cellular survival under conditions of fluctuating iron availability (18, 20).

Phosphate acquisition and homeostasis in *S. pombe* is mediated by Pho7, which is a Zn_2_Cys_6_-family transcription factor that activates the expression of phosphate-starvation response genes that house its canonical binding motif [5′-TCG(G/C)(A/T)xxTTxAA-3′] in their promoters (26–28). Under phosphate-replete conditions, upstream long non-coding RNA (lncRNA) transcription interferes with Pho7 binding to downstream PHO promoters, thereby maintaining PHO genes in a repressed state (29–32). During phosphate starvation, lncRNA-mediated blocking is relieved, which enables Pho7 to bind to its target promoters and activate phosphate transporter (*pho84+*), acid phosphatases (*pho1+*), and glycerophosphodiester transporter (*tgp1+*), in addition to activating the phosphate starvation-induced gene *ecl3+*, which is located adjacent to *pho84+* and is similarly regulated by Pho7. Thus, these events enhance phosphate acquisition and scavenging (31, 33–35). In addition to phosphate homeostasis, Pho7 functions as a multi-stress response integrator that activates the genes involved in the cellular response to copper depletion (*ctr4+*), iron deficiency (*fio1+*), osmotic stress (*gpd1+*), and glucose starvation (*hxk2+*) (34). This broader regulon suggests that Pho7 integrates signals from multiple nutrient-limiting conditions to coordinate comprehensive cellular survival strategies.

Although Fep1 and Pho7 perform central roles in nutrient homeostasis, whether they functionally interact or coordinately regulate shared target genes to implement iron–phosphate cross talk remains unknown. The activation of both phosphate-responsive and iron-responsive genes by Pho7 and the fact that both transcription factors respond to shifts in nutrient availability indicate the possibility of a functional connection between these two pathways. However, the mechanistic basis for this iron–phosphate integration mediated by these transcription factors has not been characterized.

Hence, in this study, we aimed to investigate whether Fep1 and Pho7 functionally interact to coordinate iron and phosphate homeostasis in *S. pombe*. We performed genome-wide chromatin immunoprecipitation sequencing (ChIP-seq) and RNA-sequencing (RNA-seq) analysis under controlled iron (iron-replete vs iron-depleted) and phosphate (phosphate-replete vs phosphate-starved) conditions in combination with genetic perturbations (Fep1-3sAID, Pho7-3sAID, and double mutant strains) to identify the transcriptional programs that are governed by Fep1 and Pho7 both individually and together. We detected a previously unrecognized level of coordination between iron- and phosphate-responsive transcriptional regulation and identified Fep1 and Pho7 as central components of a putative integrated nutrient-sensing network. This study provides a conceptual framework for understanding the interconnection between iron and phosphate homeostasis at the transcriptional level in fission yeast, while acknowledging that some of the observed effects may arise through indirect mechanisms rather than direct transcriptional cross-regulation.

## RESULTS

### Genome-wide identification of novel Fep1 target genes using ChIP-seq and RNA-seq

**Fig 1:**
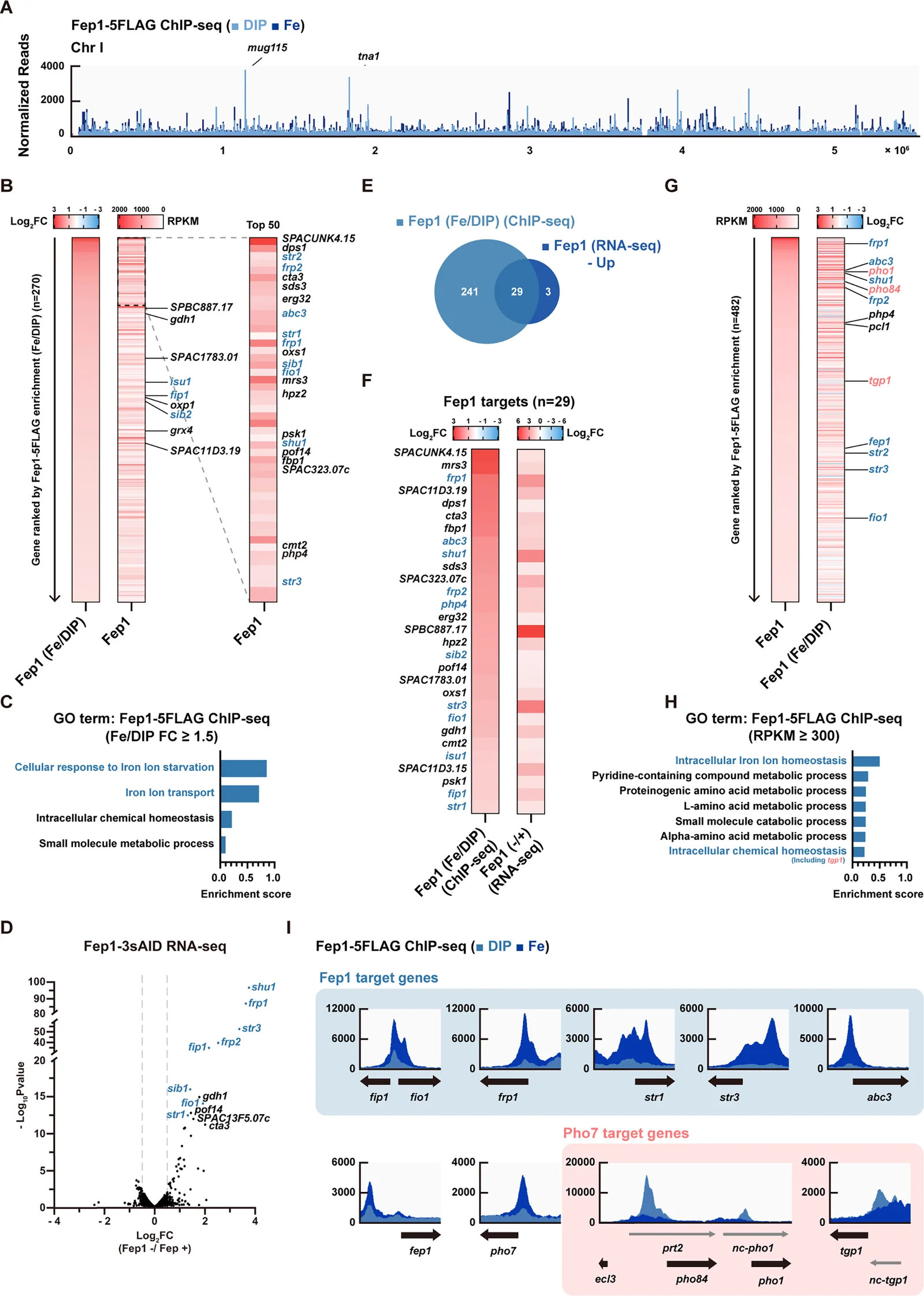
Genome-wide identification of novel Fep1 target genes using ChIP-seq and RNA-seq. (**A**) ChIP-seq profiles of Fep1-5FLAG binding across chromosome I under iron-replete (100 µM FeCl_3_) or iron-depleted (250 µM DIP) conditions. (**B**) Heat map showing Fep1-5FLAG ChIP-seq enrichment for 270 genes exhibiting ≥1.5-fold higher binding under Fe-replete vs Fe-depleted conditions. The left panel shows descending order of Log2 fold-change for Fep1-5FLAG enrichment; the middle panel shows reads per kilobase million (RPKM) values of Fep1-5FLAG under normal growth conditions; and the right panel highlights the top 50 genes ranked by RPKM. Known Fep1 targets are indicated in blue, and newly identified targets are shown in black. (**C**) Gene Ontology enrichment analysis of the 270 Fep1-bound genes shown in panel **B**. (**D**) Volcano plot showing transcriptomic changes in Fep1-3sAID cells treated with 100 nM IAA for 1 h. The *X*-axis indicates Log2 fold-change of gene expression, and *Y*-axis represents −Log10 (adjusted *P* value). Known Fep1 targets are indicated in blue, and novel targets are shown in black. (**E**) Venn diagram showing distribution of Fep1-5FLAG ChIP-seq targets (270 genes with at least 1.5-fold higher binding in Fe-replete vs Fe-depleted conditions) compared with RNA-seq results from the Fep1-3sAID degron mutant (32 genes upregulated at least 1.5-fold upon Fep1 degradation). (**F**) Heat map showing Fep1-5FLAG binding enrichment and expression levels of 29 high-affinity target genes of Fep1. The left panel shows Log2 fold-changes in Fep1-5FLAG ChIP-seq (Fe-replete vs Fe-depleted), and the right panel shows Log2 fold-changes from Fep1-3sAID RNA-seq following Fep1 degradation. (**G**) Heat map of Fep1-5FLAG ChIP-seq enrichment for 482 genes with RPKM ≥300 under basal conditions. The left panel shows Fep1-5FLAG enrichment under basal conditions, and the right panel shows Log2 fold-change of Fep1-5FLAG enrichment (Fe-replete vs Fe-depleted). Known Fep1 targets are indicated in blue, and Pho7 target genes are highlighted in pink. (**H**) Gene Ontology enrichment analysis of the 482 Fep1-bound genes identified using Fep1-5FLAG ChIP-seq (RPKM ≥300; Fig. 2A). The top enriched biological process terms are shown and ranked by enrichment score. (**I**) Fep1-5FLAG ChIP-seq peak profiles at known Fep1 target genes (*fip1+, fio1+, frp1+, str3+,* and *abc3+*) and Pho7 target genes (*pho1+, pho84+,* and *tgp1+*). Light blue indicates Fe-depleted conditions, and dark blue indicates Fe-replete conditions.

We defined the Fep1 target genes on a genome-wide scale by performing ChIP-seq using a Fep1-5FLAG epitope-tagged strain cultured under iron-replete (100 µM FeCl_3_) and iron-depleted (250 µM DIP) conditions (Fig. 1A). To confirm that C-terminal FLAG tagging did not markedly impair Fep1 function, we performed real-time quantitative PCR (RT-qPCR) analysis of the Fep1-5FLAG strain and directly compared these results with those obtained in the untagged wild-type strain. Under iron-replete conditions, *fep1+* transcript levels increased, whereas canonical iron-uptake genes were repressed in both Fep1-5FLAG and wild-type cells, consistent with the expected iron-dependent regulatory activity of Fep1 (Fig. S1A). This analysis identified 270 genes that exhibited at least 1.5-fold higher Fep1-5FLAG occupancy under iron-replete conditions than under iron-depleted conditions (Fig. 1B; Table S2A). Heat map visualization of the top 50 targets showed prominent binding at promoters of known iron uptake genes such as *fio1+*/*fip1+, frp2+*, *str1+*, *str2+*, *str3+*, and *abc3+* along with numerous previously uncharacterized loci (Fig. 1B). Gene Ontology (GO) analysis of the 270 Fep1-bound genes showed significant enrichment for terms associated with iron metabolism such as “cellular response to iron ion starvation” and “iron ion transport” (Fig. 1C; Table S2B). Volcano plot analysis showed that acute Fep1 depletion induced significant upregulation of established Fep1 target genes such as *fio1+*/*fip1+*, *frp1+*, *frp2+*, *str3+*, and *shu1+* (Fig. 1D). In total, 32 genes exhibited at least 1.5-fold increased expression upon Fep1 depletion (Table S2C).

Integration of the ChIP-seq and RNA-seq data sets helped identify 29 overlapping genes (Fig. 1E). This result provides high-confidence Fep1 targets that display both increased Fep1 binding under iron-replete conditions and elevated transcript levels upon Fep1 depletion (Fig. 1F; Table S2D). This set included well-characterized iron uptake genes (*fio1+*/*fip1+*, *frp1+*, *frp2+*, *str1+*, *str3+*, and *abc3+*) in addition to several previously uncharacterized targets such as *SPACUNK4.15, mrs3+*, *dps1+*, *cta3+*, *sds3+*, *erg32+*, *fbp1+*, *gdh1+*, and *psk1+*, which showed iron-dependent Fep1 promoter occupancy and increased transcript levels upon Fep1 depletion, indicating that they are transcriptionally iron responsive even though their roles in iron homeostasis remain to be defined. Promoter analysis of these 29 high-confidence Fep1 targets showed the presence of the canonical Fep1 binding motif (A/T)GATA(A/T) (18) within −1500 to +200 nucleotides of the transcription start site (TSS) (Fig. S2). Although the physiological relevance of the newly identified Fep1-associated candidates remains to be experimentally tested, their annotated functions suggest potential links between Fep1 regulation and broader iron-dependent cellular processes. For example, *mrs3+* encodes a mitochondrial iron carrier, consistent with the established role of Mrs3/4-type mitochondrial carriers in mitochondrial iron acquisition under iron-limiting conditions (36), whereas *dps1+* is involved in decaprenyl diphosphate synthesis for ubiquinone/coenzyme Q biosynthesis and redox metabolism (37, 38). In addition, *erg32+* is linked to sterol/ergosterol biosynthesis (39), *sds3+* is annotated as a component of the Clr6/Rpd3L histone deacetylase complex, and *psk1+* encodes a TORC1-regulated S6 kinase involved in nutrient-responsive growth control (40). These candidates may therefore represent an expanded Fep1-associated network that connects iron availability to mitochondrial function, redox metabolism, membrane homeostasis, chromatin regulation, and metabolic remodeling.

Collectively, these results show that combining ChIP-seq and RNA-seq enables robust identification of both known and previously unrecognized Fep1 target genes, which substantially expands the Fep1 regulon beyond the established iron uptake machinery.

### Fep1 target gene analysis shows unexpected binding to Pho7 and phosphate-responsive genes

After identifying Fep1 target genes based on iron-dependent changes in Fep1-5FLAG enrichment, we examined Fep1 binding independently of iron responsiveness to identify genes that are robustly and strongly occupied by Fep1 under basal conditions (reads per kilobase million [RPKM] ≥300). We ranked the genes according to their basal Fep1-5FLAG ChIP-seq occupancy, which was measured using the RPKM values rather than the iron-dependent fold changes. This analysis unexpectedly showed that Fep1 bound not only to the canonical iron-regulated genes but also to the *pho7+* locus and multiple Pho7-regulated phosphate-responsive genes. In total, 482 Fep1-bound genes were identified (RPKM ≥300), including the canonical Pho7 target genes *pho1+*, *pho84+*, and *tgp1+*, which are highlighted in pink in the heat map in Fig. 1G and Table S2E. GO enrichment analysis of these 482 genes identified “Intracellular iron ion homeostasis,” which included established iron-related genes, as the most significantly enriched category in addition to “Intracellular chemical homeostasis,” which included the Pho7 target gene *tgp1+* (Fig. 1H; Table S2F). Owing to the unexpected enrichment of Pho7 target genes among Fep1-bound loci, we examined whether Fep1 binding to these phosphate-responsive promoters exhibited iron-dependent regulation.

Heat map visualization of both absolute RPKM values and iron-dependent fold changes (Fe +/− FC) indicated that Fep1 occupancy at several Pho7 target genes is itself dynamically regulated by iron availability (Fig. 1G). Furthermore, we characterized these iron-dependent binding patterns at individual loci by inspecting the Fep1-5FLAG ChIP-seq profiles using the Integrative Genomics Viewer (IGV) (Fig. 1I). We found that Fep1 occupancy was elevated at the *pho7+* promoter under iron-replete conditions, which was consistent with the canonical Fep1 function. In contrast, Fep1 binding at Pho7 target genes (*pho1+, pho84+,* and *tgp1+*) increased under iron-depleted conditions. This reciprocal binding behavior indicates that a subset of Pho7-responsive promoters is associated with Fep1 under iron-depleted conditions, despite lacking canonical Fep1 GATA-type motifs within the detected Fep1-bound regions. Thus, Fep1 occupancy at these loci appears distinct from its classical binding pattern at iron-uptake genes and may reflect noncanonical or indirect promoter association.

### Pho7 reciprocally associates with Fep1 target gene subset and regulates their expression

**Fig 2:**
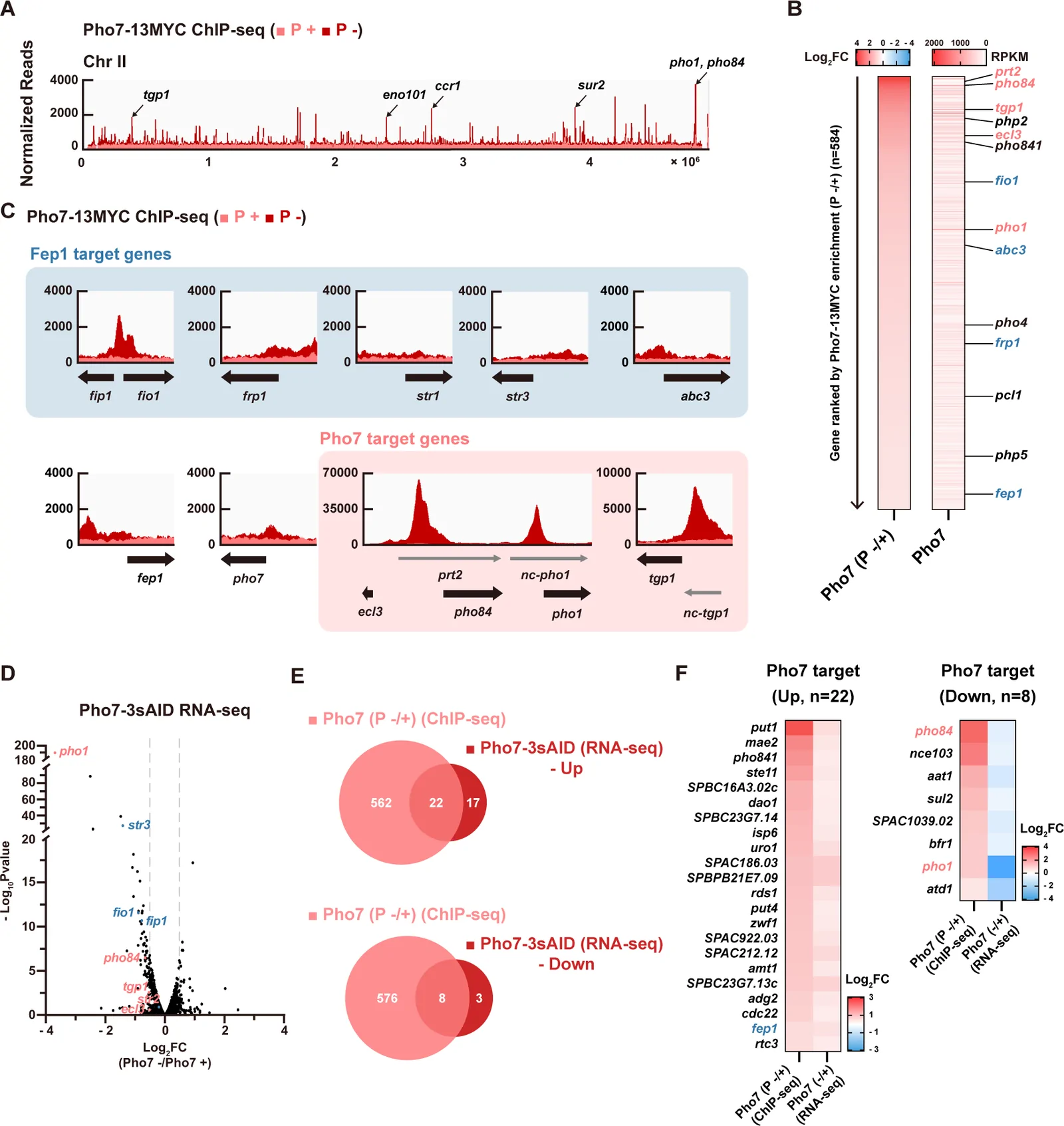
Genome-wide identification of Pho7 target genes indicates extensive overlap with Fep1 targets. (**A**) Pho7-13MYC ChIP-seq profiles across chromosome I under phosphate-replete (P +) and phosphate-starvation (P −) conditions. Cells were grown in EMM medium and transferred to either EMM (P +) or EMM-P (P −) medium for 4 h before ChIP-seq analysis. (**B**) Heat map of Pho7 ChIP-seq enrichment for 334 genes showing ≥1.5-fold increased Pho7 binding under phosphate starvation relative to phosphate-replete conditions. The left panel shows Log2 fold-change values (P −/+), and the right panel shows RPKM values under basal conditions. Pho7 targets are marked in pink, and Fep1 targets are indicated in blue. (**C**) Pho7-13MYC ChIP-seq peak profiles at Fep1 target genes (*fio1+*, *fip1+*, *frp1+*, *str3+*, and *abc3+*) and Pho7 target genes (*pho84+*, *pho1+*, and *tgp1+*). Light pink indicates phosphate-replete conditions, and red indicates phosphate-starvation conditions. (**D**) Volcano plot showing transcriptomic changes in Pho7 degron mutants treated with 100 nM IAA for 1 h. The *X*-axis indicates Log2 fold-change in gene expression, and the *Y*-axis represents −Log_10_ (adjusted *P* value). Known Pho7 targets are shown in pink, and Fep1 target genes are shown in blue. (**E**) Venn diagrams comparing Pho7-13MYC targets with differentially expressed genes identified using RNA-seq in Pho7-3sAID cells. Overlap between Pho7-13MYC ChIP-seq targets with ≥1.5-fold increased binding under phosphate starvation (P −/+; *n* = 584) and genes upregulated (left; *n* = 39) or downregulated (right; *n* = 11) upon Pho7 depletion. (**F**) Heat map showing Pho7-13MYC ChIP-seq binding enrichment and RNA-seq expression profiles upon Pho7 depletion. Left heat map: genes upregulated upon Pho7 degradation (*n* = 22). Right heat map: genes downregulated upon Pho7 degradation (*n* = 8). In each heat map, the left panel shows Log2 fold-change of Fep1-5FLAG ChIP-seq enrichment (phosphate-replete vs phosphate-depleted), and the right panel displays Log2 fold-changes in gene expression from Pho7-3sAID RNA-seq after Pho7 degradation. Known Pho7 targets are highlighted in pink, and established Fep1 targets are highlighted in blue.

We assessed whether Pho7 reciprocally associates with Fep1 target genes by performing ChIP-seq analysis using a Pho7-13MYC epitope-tagged strain cultured under phosphate-replete and phosphate-depleted conditions (Fig. 2A). To verify that C-terminal MYC tagging did not markedly impair Pho7 function, we performed RT-qPCR analysis of the Pho7-13MYC strain and directly compared these results with those obtained in the untagged wild-type strain. Under phosphate-starved conditions, *pho7+* transcript levels were largely unchanged, whereas canonical Pho7 target genes, including *pho1+*, *pho84+*, *tgp1+* and *ecl3+*, were robustly induced in both Pho7-13MYC and wild-type cells, consistent with the expected phosphate-responsive activity of Pho7 (Fig. S1B). Phosphate starvation was induced by culturing cells in phosphate-free Edinburgh Minimal Medium (EMM-P) for 4 h. This analysis identified 584 genes that exhibited at least 1.5-fold increased Pho7 binding under phosphate-depleted conditions. The identified genes included *fep1+* itself and Fep1 target genes such as *fio1+*/*fip1+*, *abc3+*, and *frp1+* (Fig. 2B; Table S3A). Inspection of Pho7-13MYC ChIP-seq profiles using the IGV showed increased Pho7 occupancy at multiple Fep1 target promoters under phosphate-depleted conditions. Particularly, prominent enrichment was identified at the *fio1+* and *fip1+* loci with modest increases noticed at additional Fep1-regulated genes (Fig. 2C). These observations indicate that Pho7 directly associates with a subset of Fep1 target promoters in a phosphate-dependent manner.

GO analysis of Pho7-13MYC ChIP-seq data indicated distinct functional categories based on the gene set that was analyzed. GO enrichment analysis of the 278 genes with high basal Pho7 occupancy (RPKM ≥300) showed that the primary enriched term was “Induction of conjugation with cellular fusion” (Fig. S3A; Table S3B). We investigated the functional landscape of Pho7-regulated genes by expanding the analysis to a larger gene set defined by phosphate-responsive Pho7 binding (P −/+ FC ≥ 1.5; *n* = 584). We found enrichment for biologically relevant categories, including “Amino acid catabolic processes,” “Small-molecule metabolic processes,” and “Transmembrane transport,” which encompassed both canonical Pho7 targets (*pho84+* and *tgp1+*) and Fep1 targets (*abc3+* and *frp1+*) (Fig. S3B; Table S3C).

We determined whether Pho7 binding at these loci results in transcriptional activation by performing RNA-seq using a Pho7-3sAID degron strain. We found that the expression of Fep1 target genes *fio1+*, *fip1+*, and *str3+* decreased in the absence of Pho7 (Fig. 2D), which indicated that Pho7 contributes to the transcriptional output of these genes (Tables S3D and E). Integration of the Pho7-13MYC ChIP-seq and Pho7-3sAID RNA-seq data sets helped identify two overlapping gene sets that included 22 upregulated and 8 downregulated genes under the Pho7-depleted condition (Fig. 2E). Complete lists of these genes are provided in Fig. 2F and Table S3F and G. Furthermore, we characterized this gene set by examining the promoter regions located −1500 to +200 nucleotides relative to the TSS of the 22 upregulated and 8 downregulated genes under Pho7-depleted condition (Fig. S3A and B). Promoter analysis was performed separately for the two overlapping gene sets. We found that the Fep1 binding motif (A/T)GATA(A/G) was present in 16 of the 22 promoters of the 22 upregulated genes that were identified under Pho7-depleted condition, whereas the known Pho7 binding motif TCG(G/C)(A/T)xxTTxAA (26–28) was only detected in 3 of the 22 promoters (Fig. S4A). In contrast, the Fep1 binding motif was identified in five of the eight promoters of the eight genes that were downregulated upon Pho7 depletion; additionally, the canonical Pho7 binding motif was present in five of the eight promoters (Fig. S4B). Next, we performed unbiased motif discovery on the eight Pho7-dependent downregulated targets using MEME-ChIP and scored the resulting motifs with the STREAM tool. This analysis identified two enriched sequence motifs 5′-CWTCTTAA′-3′ and 5′-YTGACA-3′ with *E* values of 7.00 × 10^−4^, respectively (Fig. S4C). This result provides independent support for conserved cis-regulatory elements within this subset of Pho7 targets. Together, these reciprocal binding and expression patterns indicate that Pho7 directly regulates a subset of Fep1 target genes. This inference highlights an unexpected regulatory intersection between iron- and phosphate-responsive transcriptional programs in *S. pombe*.

### Fep1 restricts Pho7 chromatin binding, and Pho7 supports basal expression of Fep1 target genes

**Fig 3:**
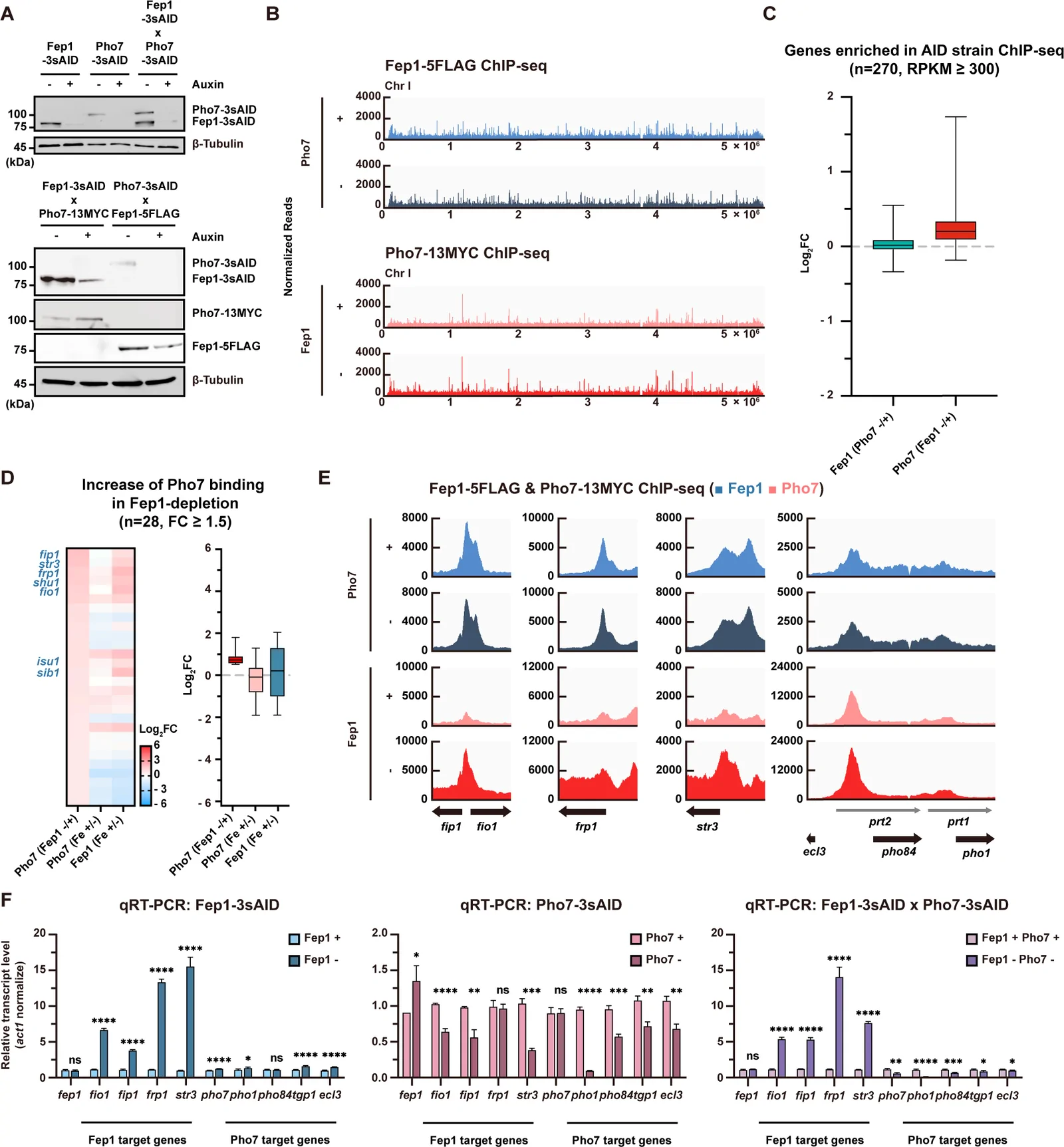
Pho7 binding to Fep1 target promoters is enhanced under Fep1 absence. (**A**) Western blotting analysis of Fep1 and Pho7 protein levels following auxin-induced degradation. Upper panel: Fep1-3sAID, Pho7-3sAID, and Fep1-3sAID × Pho7-3sAID. Lower panel: Fep1-3sAID × Pho7-13MYC and Pho7-3sAID x Fep1-5FLAG. Cells were treated with 100 nM auxin for 1 h to induce Fep1 and Pho7 degradation. (**B**) Comparison of Fep1-5FLAG and Pho7-13MYC ChIP-seq binding on chromosome I under conditions of Pho7 or Fep1 depletion. Upper tracks: Fep1-5FLAG ChIP-seq profiles in the presence (+) or absence (−) of Pho7. Lower tracks: Pho7-13MYC ChIP-seq profiles in the presence (+) or absence (−) of Fep1. (**C**) Box plots comparing Fep1-5FLAG and Pho7-13MYC binding changes at the 270 co-occupied target genes (RPKM ≥300 in both ChIP-seq data). Fep1-5FLAG binding shows minimal change upon Pho7 depletion (Fep1 [Pho7 −/+]), whereas Pho7-13MYC binding increased substantially upon Fep1 depletion (Pho7 [Fep1 −/+]). (**D**) Analysis of Pho7 binding changes upon Fep1 degradation. Left heat map shows Log2 fold-change values for 28 genes with at least 1.5-fold increased Pho7-13MYC binding in Fep1-degraded cells, compared across multiple conditions: Pho7 (Fep1 −/+), Pho7 (P −/+), Pho7 (Fe/DIP), and Fep1 (Fe/DIP). Right box plots quantify ChIP-seq enrichment under the same conditions. Additional categories shown are increased Fep1 binding upon Pho7 deletion (*n* = 1), decreased Fep1 binding upon Pho7 deletion (*n* = 0), and decreased Pho7 binding upon Fep1 deletion (*n* = 1). (**E**) Fep1-5FLAG and Pho7-13MYC ChIP-seq peak profiles at selected Fep1 target genes (*fip1+*, *fio1+*, *frp1+*, and *str3+*) and select Pho7 target genes (*pho84*+ and *pho1+*) from the 28 genes identified in panel **D**. Pho7-13MYC binding increased at Fep1 target genes upon Fep1 deletion, whereas Fep1-5FLAG binding remains low or absent under Pho7 deletion. (**F**) RT-qPCR analysis of Fep1 and Pho7 target gene expression in Fep1-3sAID, Pho7-3sAID, and Fep1-3sAID × Pho7-3sAID strains following auxin-induced factor degradation (100 nM IAA for 1 h). Left panel: Fep1-3sAID strain showing transcript levels of Fep1 targets and Pho7 targets. Middle panel: Pho7-3sAID strain showing corresponding transcript changes. Right panel: Fep1-3sAID × Pho7-3sAID double mutant showing effects of simultaneous Fep1 and Pho7 depletion. Transcript levels were normalized to that of *act1+*. Data represent the mean ± SD from three independent biological experiments. Statistical significance: ****P < 0.0001; ***P < 0.001; **P < 0.01; *P < 0.05; and ns, not significant.

We examined the effects of acute depletion of each transcription factor on genome-wide binding patterns by performing degradation experiments using the auxin-inducible degron (AID) system. Western blotting analysis confirmed the efficient depletion of both Fep1 and Pho7 upon treatment with 100 nM IAA for 1 h (Fig. 3A). In single-tagged strains that expressed either Fep1-3sAID or Pho7-3sAID, the corresponding protein was rapidly degraded, whereas simultaneous depletion of both factors was observed in the double-tagged strain (Fep1-3sAID × Pho7-3sAID). This finding validates the specificity and efficiency of the AID system. To assess whether depletion of one factor affected the abundance of the other, we introduced Fep1-5FLAG and Pho7-13MYC epitope tags into the AID strains and performed immunoblotting analysis. No apparent differences were observed in Pho7-13MYC or Fep1-5FLAG protein levels under depletion conditions, which indicates that acute depletion of one factor did not measurably affect the abundance of the other at the protein level (Fig. 3A).

Genome-wide ChIP-seq analysis showed a pronounced asymmetry in the reciprocal regulation of Fep1-5FLAG and Pho7-13MYC chromatin binding. Pho7 depletion resulted in minimal changes in Fep1-5FLAG occupancy across the genome, which indicated that Pho7 does not substantially contribute to the control of Fep1 chromatin association (Fig. 3B). In contrast, Fep1 depletion substantially increased Pho7-13MYC occupancy. Particularly, strong enhancement was observed at loci that already exhibited high Pho7 binding. We determined whether this Fep1-dependent regulation of Pho7 binding extended across a broader set of shared targets by quantifying the binding fold changes for the 215 genes that exhibited enrichment of both Fep1-5FLAG and Pho7-13MYC. Across this gene set, Fep1-5FLAG occupancy remained largely unchanged after Pho7 depletion, which supports a minimal role for Pho7 in regulating Fep1 binding. In contrast, Pho7-13MYC occupancy was consistently elevated upon Fep1 depletion (Fig. 3C; Table S4A). Together, these findings show that Fep1 depletion selectively enhances Pho7 chromatin occupancy at shared target sites. This finding indicated an unanticipated role for Fep1 in limiting Pho7 binding.

We determined the genes that exhibited increased Pho7 binding upon Fep1 loss loci by identifying the genes that showed ≥1.5-fold increase in Pho7-13MYC occupancy after Fep1 depletion (Fig. 3D; Table S4B). Notably, several of these genes corresponded to previously characterized iron-responsive loci, including *fip1+*, *str3+*, *frp1+*, *shu1+*, *fio1+*, *isu1+*, and *sib1+*. This gene set was enriched for loci at which Fep1-5FLAG occupancy was elevated under iron-replete conditions. To validate these genome-wide trends, we examined the representative IGV tracks for selected Fep1 target genes (*fip1+*, *fio1+*, *frp1+*, and *str3+*), with Pho7 target genes (*pho1+*, *pho84+*, and *ecl3+*) included as comparative controls (Fig. 3E). Consistent with the result of the global ChIP-seq analysis, Pho7 depletion exerted little effect on Fep1-5FLAG occupancy at these loci, whereas Fep1 depletion resulted in a pronounced increase in Pho7-13MYC signal. These observations indicate that Fep1 functions to antagonize Pho7 chromatin binding at a subset of its primary target loci.

We determined whether altered Pho7 chromatin occupancy translated into changes in gene expression by examining transcript levels using RT-qPCR after acute depletion of Fep1 and/or Pho7 (Fig. 3F). Under Fep1-depleted condition, Fep1 target genes showed upregulation, which was consistent with the loss of Fep1-mediated transcriptional repression. In contrast, the expression of Pho7 target genes was largely unaffected, which indicates that Fep1 did not exert a dominant transcriptional influence over the phosphate-responsive regulon. Pho7 depletion resulted in a distinct transcriptional response. As expected, Pho7 target genes were downregulated because of the absence of Pho7-dependent activation. Notably, a subset of Fep1 target genes that included *fio1+*, *fip1+*, and *str3+* additionally exhibited reduced transcript levels under Pho7-depleted conditions, with *fio1+* at 0.62-fold, *fip1+* at 0.57-fold, and *str3+* at 0.37-fold relative to the control (Fig. 3F). These genes corresponded to loci at which Pho7 binding was enhanced following Fep1 depletion (Fig. 3D and E). This indicated that Pho7 contributed to the basal expression of specific Fep1 target genes. In the double-degron strain, simultaneous depletion of Fep1 and Pho7 did not result in synergistic transcriptional effects. Instead, transcriptional outcomes were largely dictated by the dominant regulatory factor for each gene class. Fep1 target genes, such as *fio1+*, *fip1+*, *frp1+*, and *str3+*, showed strong induction, reaching approximately 5.0-, 4.6-, 13.1-, and 7.4-fold of the control levels, respectively, representing an overall upregulation consistent with the removal of Fep1-mediated repression. In contrast, Pho7 target genes, such as *pho1+*, *pho84+*, *tgp1+*, and *ecl3+*, were reduced to about 0.04-, 0.56-, 0.75-, and 0.85-fold, respectively (Fig. 3F).

In addition, RT-qPCR analysis of representative Fep1 and Pho7 target genes under controlled iron (iron-replete vs iron-depleted) and phosphate (phosphate-replete vs phosphate-starved) conditions confirmed the expected nutrient-dependent changes in their transcript levels. For Fep1 target genes (*fio1+*, *fip1+*, *frp1+*, *str1+*, and *str3+*), iron depletion, but not phosphate starvation, led to marked induction (Fig. S1A and S5A). Conversely, Pho7 target genes (*pho1+*, *pho84+*, *tgp1+*, and *ecl3+*) were robustly induced by phosphate starvation (Fig. S1B and S5B). Under iron-depleted conditions, *pho84+* and *tgp1+* showed a modest reduction in transcript levels, coinciding with increased Fep1 occupancy at these promoters in Fig. 1I, whereas *pho1+* expression was not clearly affected by iron availability.

Taken together, these results indicate that in addition to their primary and largely independent regulatory roles, Pho7 contributes to sustaining the basal expression of a subset of Fep1 target genes. This functional contribution becomes evident upon Fep1 loss. This finding provides a mechanistic link between iron-dependent Fep1 regulation and Pho7-mediated transcriptional activation.

### Iron-dependent transcriptional outputs of Fep1 and Pho7 and their impact on cellular fitness

Next, we examined the mechanisms underlying the translation of these regulatory interactions into transcriptional outcomes under distinct iron conditions. We used the AID system to acutely deplete Fep1 and/or Pho7 (IAA treatment for 1 h) under iron-depleted and iron-replete conditions. Then, the transcript levels of the representative genes involved in iron uptake and phosphate homeostasis were quantified using RT-qPCR.

**Fig 4:**
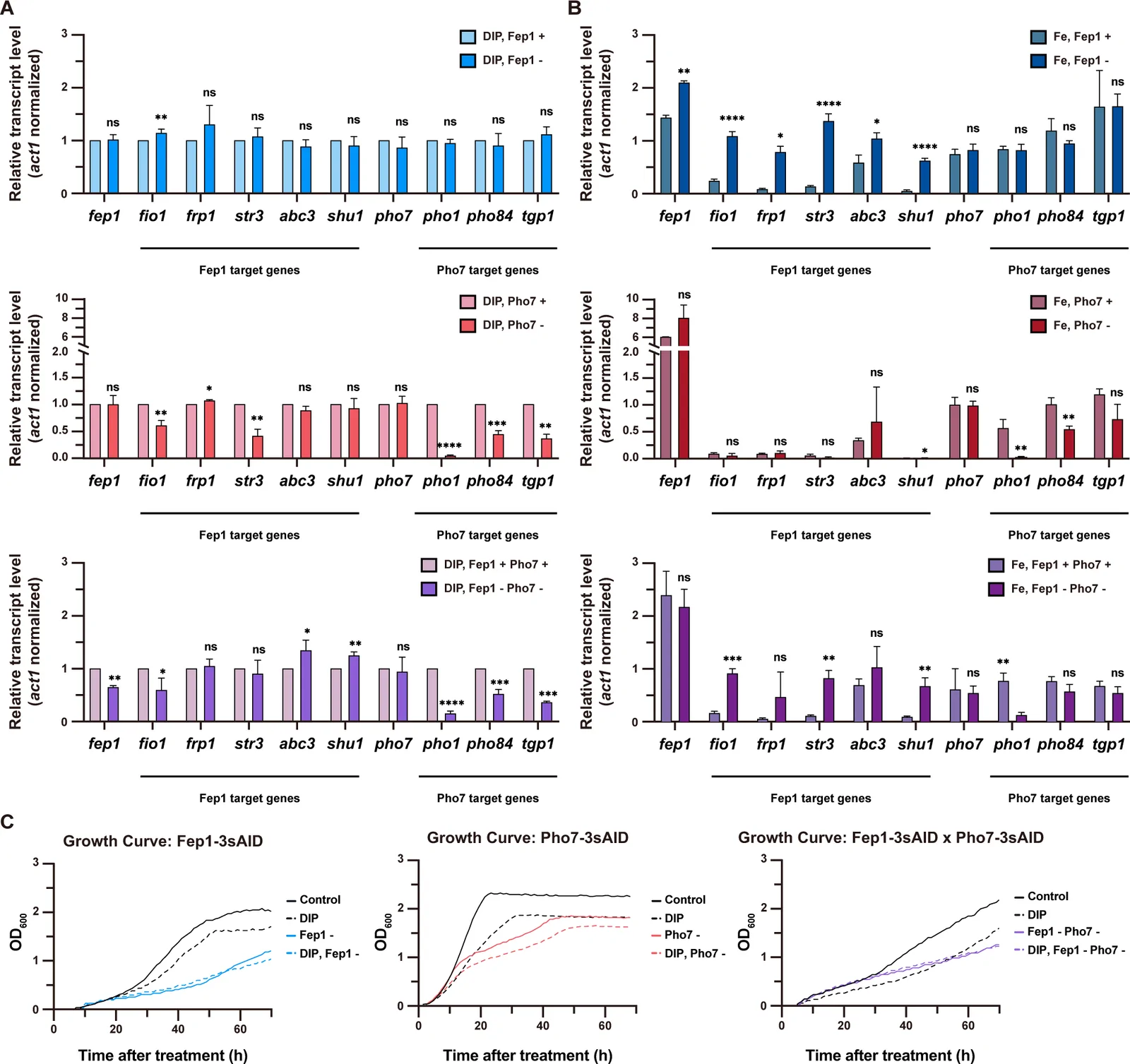
Integration of canonical and cross-regulatory functions of Fep1 and Pho7. (**A**) Quantitative RT-PCR analysis of Fep1 target genes (iron-uptake genes: *fio1+*, *frp1+*, *str3+*, *abc3+*, and *shu1+*) and Pho7 target genes (PHO pathway genes: *pho1+*, *pho84+*, and *tgp1+*) upon depletion of Fep1, Pho7, or both factors under iron-depleted conditions (250 μM DIP, 1.5 h). Row 1, Fep1-3sAID strain (Fep1 +/−); row 2, Pho7-3sAID strain (Pho7 +/−); and row 3, Fep1-3sAID × Pho7-3sAID double mutant (Fep1 +/−, Pho7 +/−). Cells were treated with 100 nM IAA for 1 h to induce Fep1 and Pho7 degradation. Transcript levels were normalized to that of *act1+*. Data represent the mean ± SD from three independent biological experiments. Statistical significance: ****P < 0.0001; ***P < 0.001; **P < 0.01; *P < 0.05; and ns, not significant. (**B**) Quantitative RT-PCR analysis of Fep1 target genes (iron-uptake genes: *fio1+*, *frp1+*, *str3+*, *abc3+*, and *shu1+*) and Pho7 target genes (PHO pathway genes: *pho1+*, *pho84+*, and *tgp1+*) upon depletion of Fep1, Pho7, or both factors under iron-replete conditions (100 μM FeCl_3_, 1.5 h). Row 1, Fep1-3sAID strain (Fep1 +/−); row 2, Pho7-3sAID strain (Pho7 +/−); and row 3, Fep1-3sAID × Pho7-3sAID double mutant (Fep1 +/−, Pho7 +/−). Cells were treated with 100 nM IAA for 1 h to induce Fep1 and Pho7 degradation. Transcript levels were normalized to that of *act1+*. Data represent the mean ± SD from three independent biological experiments. Statistical significance: ****P < 0.0001; ***P < 0.001; **P < 0.01; *P < 0.05; and ns, not significant. (**C**) Growth curves of Fep1-3sAID, Pho7-3sAID, and Fep1-3sAID × Pho7-3sAID strains under iron-depleted conditions. Cells were cultured to an OD_600_ of 0.5, followed by treatment with IAA (100 nM), DIP (50 μM), or both. Control cells were treated with DMSO only. Growth was monitored for 80 h.

Fep1 depletion resulted in distinct transcriptional outcomes depending on iron availability. Under iron-depleted conditions, Fep1 loss showed negligible impact on the expression of its canonical target genes, which was consistent with Fep1 being inactive as a repressor at low cellular iron concentrations (Fig. 4A). In contrast, under iron-replete conditions, Fep1 depletion led to pronounced upregulation of Fep1 target genes, which confirmed its role as a repressor in high-iron environments (Fig. 4B). Importantly, Fep1 depletion did not significantly alter the expression of Pho7 target genes regardless of iron status, which indicated that Fep1 did not directly regulate the phosphate-responsive regulon.

In contrast, Pho7 depletion resulted in increased transcriptional changes. Under iron-depleted conditions, the PHO pathway genes showed consistently decreased transcript levels. Notably, Pho7 depletion among the Fep1 target genes that showed increased Pho7 binding upon Fep1 degradation (e.g., *fio1+* and *str3+*) led to reduced mRNA abundance (*fio1+* to approximately ~0.61-fold; *str3+* to approximately ~0.41-fold) (Fig. 4A). Under iron-replete conditions, Pho7 depletion resulted in a distinct decrease in the expression of Pho7 target genes such as *pho1+* and *tgp1+*, which was consistent with the loss of Pho7-dependent transcriptional activation. In contrast, the expression of Fep1 target genes was largely unaffected by Pho7 depletion under these conditions (Fig. 4B). This was consistent with the high abundance and dominant repressive activity of Fep1 in iron-replete environments, in which Fep1-mediated repression overrides potential contributions from Pho7. Accordingly, Pho7 presence or absence elicited minimal impact on the transcriptional output of Fep1 target genes at insufficient iron levels.

In the double-degron strain (Fep1-3sAID × Pho7-3sAID), simultaneous depletion of both factors did not result in synergistic effects; rather, the transcriptional pattern of each gene set was primarily driven by its dominant regulatory factor, which reiterated the trends observed in single degron strains (Fig. 4A and B).

We assessed the physiological relevance of these transcriptional changes by examining the impact of transcription factor depletion on cell growth under iron-limiting conditions (Fig. 4C). In the Fep1-3sAID strain, OD_600_ measurements at 60 h revealed clear growth impairment, with the control culture reaching 1.99, whereas Fep1 depletion reduced OD_600_ values to 0.93 (≈47% of the control) and iron starvation reduced it to 1.61 (≈81% of the control). By contrast, in the Pho7-3sAID strain, the control culture reached an OD_600_ of 3.03, which decreased to 2.42 upon Pho7 depletion (≈80% of the control) and to 2.44 under iron-limiting conditions (≈81% of the control), while the combination of Pho7 depletion and iron limitation reduced OD_600_ further to 2.17 (≈72% of the control). These results indicate that Pho7 depletion caused a modest additional reduction in growth under iron-limiting conditions. Therefore, Pho7-dependent transcription may contribute to cellular fitness under iron scarcity, although this physiological effect appears limited in magnitude under the conditions tested. The double-degron strain (Fep1-3sAID × Pho7-3sAID) exhibited substantial growth impairment irrespective of iron availability, which precluded the assessment of additive growth defects specifically under iron-depleted conditions.

Collectively, these results establish Pho7 as a key determinant of cellular fitness under iron-limiting conditions. Under iron starvation, Pho7 supports the basal expression of a subset of iron uptake genes such as *fio1+*, *fip1+*, and *str3+*. Additionally, the loss of Pho7 compromises growth under these conditions.

### Iron availability modulates shared chromatin binding by Fep1 and Pho7

We systematically characterized the overlap of Fep1 and Pho7 binding sites under all combinations of iron and phosphate conditions. First, we examined the expression and localization of Fep1-5FLAG and Pho7-13MYC proteins (Fig. S6A and B). Fep1-5FLAG protein levels decreased under iron-depleted conditions, which was consistent with known Fep1 regulation. In contrast, Pho7-13MYC protein levels remained largely unchanged under both iron and phosphate conditions (Fig. S6A). Second, we assessed the subcellular localization of both transcription factors using Fep1-GFP and Pho7-mCherry strains. Regardless of iron and phosphate availability, both Fep1-GFP and Pho7-mCherry showed consistent localization to the nucleus, and their signal intensities were comparable as well (Fig. S6B).

**Fig 5:**
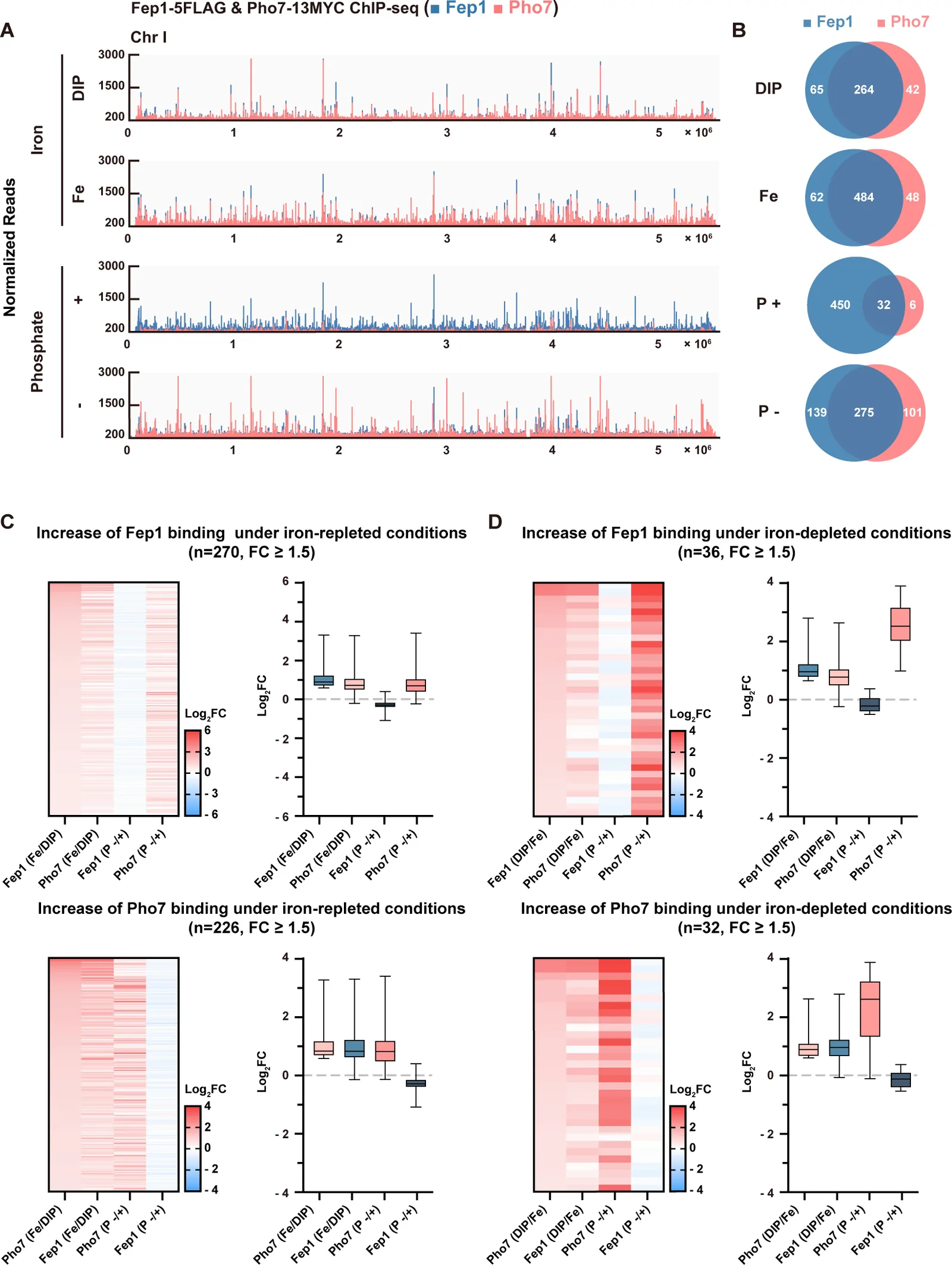
Extensive co-occupancy of Fep1 and Pho7 at shared target loci with nutrient-dependent binding patterns. (**A**) Genome-wide ChIP-seq profiles of Fep1-5FLAG and Pho7-13MYC across chromosome I under iron-replete (Fe) and phosphate-starvation (P −) conditions. Fep1-5FLAG binding is indicated in blue, and Pho7-13MYC binding is indicated in pink. Numerous genomic regions show overlapping occupancy by both factors. (**B**) Venn diagram showing the overlap between Fep1- and Pho7-bound genes (RPKM ≥300) under four different conditions (DIP, Fe, P +, and P −). Fep1-bound genes are indicated in blue, and Pho7-bound genes are in pink. (**C**) Analysis of Fep1 and Pho7 co-occupancy under iron-replete conditions. Upper panels show heat maps and box plots comparing Pho7 binding across different conditions at genes with ≥1.5-fold increased Fep1 binding under iron-replete conditions (*n* = 270). Lower panels show heat maps and box plots comparing Fep1 binding across different conditions at genes with ≥1.5-fold increased Pho7 binding under iron-replete conditions (*n* = 226). These results indicate that genes with elevated Fep1 binding under iron-replete conditions additionally show elevated Pho7 binding. Similarly, genes with elevated Pho7 binding show elevated Fep1 binding, which indicates substantial Fep1 and Pho7 co-occupancy at shared target genes. (**D**) Analysis of Fep1 and Pho7 co-occupancy under iron-depleted conditions. Upper panels show heat maps and box plots comparing Pho7 binding across different conditions at genes with ≥1.5-fold increased Fep1 binding under iron-depleted conditions (*n* = 36). Lower panels show heat maps and box plots comparing Fep1 binding across different conditions at genes with ≥1.5-fold increased Pho7 binding under iron-depleted conditions (*n* = 32). In concurrence with the results shown for iron-replete conditions shown in panel **C**, genes with elevated Fep1 binding under iron-depleted conditions additionally show elevated Pho7 binding. Similarly, genes with elevated Pho7 binding show elevated Fep1 binding. These results indicate that the co-occupancy pattern of Fep1 and Pho7 is maintained under both iron-replete and iron-depleted conditions, which establish an iron-independent coordination of Fep1 and Pho7 binding at shared target loci.

Third, we performed ChIP-seq analysis using Fep1-5FLAG and Pho7-13MYC strains under all combinations of iron and phosphate conditions. In concurrence with previously reported results (34, 41), the genome-wide binding patterns of each factor showed strong condition dependency (Fig. 5A). Fep1 exhibited globally increased chromatin association under iron-replete conditions, which was consistent with its established role as an iron-responsive repressor, whereas Pho7 binding substantially enhanced under phosphate-depleted conditions. Notably, Pho7 occupancy decreased significantly under phosphate-replete conditions due to an overall attenuation of Pho7 binding across the genome. Despite these distinct and well-characterized regulatory patterns, comparison of binding profiles along chromosome I showed extensive regions of overlapping occupancy between the two transcription factors (Fig. 5A). We quantified this overlap by comparing the genes exhibiting RPKM values of ≥300 for either Fep1-5FLAG or Pho7-13MYC under different conditions. This analysis identified 264 overlapping genes under iron-depleted, 484 under iron-replete, 32 under phosphate-replete, and 275 under phosphate-depleted conditions (Fig. 5B; Tables S5A–D). The low overlap observed under phosphate-replete conditions reflected the pronounced decrease in Pho7 binding as only 38 Pho7-bound genes satisfied the RPKM threshold. Overall, these results indicate that despite their distinct nutrient-responsive binding behaviors, Fep1 and Pho7 co-occupy a substantial number of genomic loci under most environmental conditions.

Next, we investigated the role of iron availability in this overlap by examining iron-dependent changes in Fep1 and Pho7 binding at shared target genes using ChIP-seq data (Fig. 5C and D). Notably, increased Fep1 binding at iron-responsive targets was frequently accompanied by a corresponding increase in Pho7 occupancy. Under iron-replete conditions, where Fep1 is highly expressed and actively represses iron uptake genes, previous ChIP-seq analysis identified 270 genes that exhibited ≥1.5-fold increased Fep1-5FLAG binding (Fig. 1B; Table S2A). Analysis of Pho7-13MYC ChIP-seq data showed that Pho7 binding was additionally elevated at the majority of these loci under iron-replete conditions (Fig. 5C; Table S5E), which indicated the coordinated recruitment of the two factors at iron-responsive targets.

Generally, Fep1 acts predominantly under iron-replete conditions; nevertheless, we observed that a small subset of genes exhibited increased Fep1-5FLAG binding under iron-depleted conditions. Specifically, 36 genes showed a ≥1.5-fold increase in Fep1-5FLAG occupancy under iron depletion. Notably, Pho7-13MYC binding increased at the majority of these loci, which indicated the coordinated recruitment of Pho7 to genes that showed enhanced Fep1 binding under low-iron conditions (Fig. 5D; Table S5F). Promoter analysis of these iron-depletion-responsive Fep1 targets was refined to focus on chromatin occupancy and *de novo* motif discovery. Specifically, we highlighted Fep1-5FLAG and Pho7-13MYC enrichment peaks within the −1500 to +200 nucleotide regions surrounding the transcription start sites and then selected 25 genes that displayed clear Fep1-5FLAG peaks in their promoters for motif analysis. Using MEME-ChIP followed by scoring with the STREAM tool, we identified three enriched sequence motifs: 5′-AGCTCT-3′ (*E* value, 1.60 × 10^−4^), 5′-AATTGGTMK-3′ (*E* value, 3.40 × 10^−4^), and 5′-GCCTAT-3′ (*E* value, 5.10 × 10^−4^). These findings raise the possibility that apo-Fep1 under iron-depleted conditions may recognize distinct cis-regulatory motifs, different from the canonical holo-Fep1 motif, when binding to this set of targets, although the precise mechanistic basis for apo-Fep1 binding remains unresolved and will require further experimental investigation (Fig. S7). In contrast, Pho7-13MYC binding increased ≥1.5-fold in 32 genes under iron-depleted conditions, and Fep1-5FLAG binding was likewise elevated at most of these loci (Fig. 5D; Table S5G). Together, these reciprocal patterns indicate that even under iron-depleted conditions, increased binding of one factor is frequently accompanied by enhanced association of the other at a shared subset of target genes.

### Iron availability determines the hierarchy of Fep1 and Pho7 in cross-regulation of target genes

We found that iron availability strongly influenced the extent of Fep1–Pho7 co-occupancy at shared genomic loci. Therefore, we determined the manner in which the two transcription factors hierarchically regulated transcription at these shared targets by identifying the specific target genes that were jointly bound by Fep1 and Pho7 under different iron conditions.

**Fig 6:**
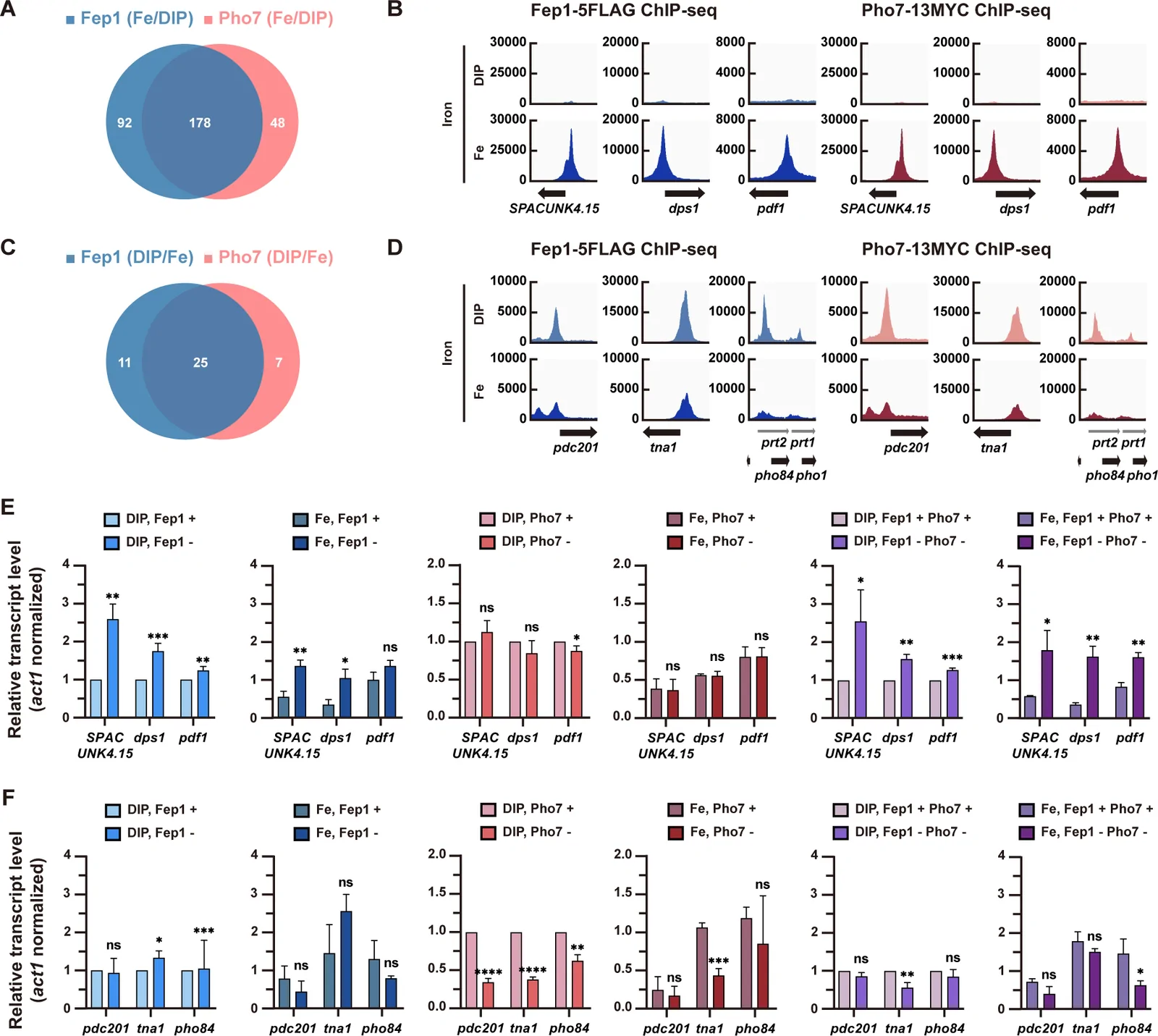
Iron availability differentially regulates Fep1 and Pho7 co-occupancy and transcriptional outputs at shared target genes. (**A**) Venn diagram showing overlap of Fep1 and Pho7 target genes (≥1.5-fold increased binding) under iron-replete conditions. (**B**) ChIP-seq profiles of three co-occupied genes (*SPACUNK4.15, dps1+*, and *pdf1+*) under iron-depleted and iron-replete conditions. Both Fep1-5FLAG (blue) and Pho7-13MYC (pink) show increased binding under iron-replete conditions. (**C**) Venn diagram showing overlap of Fep1 and Pho7 target genes (≥1.5-fold increased binding) under iron-depleted conditions. (**D**) ChIP-seq profiles of three co-occupied genes (*pdc201+*, *tna1+*, and *pho84+*) under iron-depleted and iron-replete conditions. Both Fep1-5FLAG and Pho7-13MYC show increased binding under iron-depleted conditions. (**E**) Quantitative RT-PCR analysis of iron-replete target genes (*SPACUNK4.15, dps1+*, and *pdf1+*) upon depletion of Fep1, Pho7, or both factors under iron-replete and iron-depleted conditions. Rows 1 and 2, Fep1-3sAID strain (Fep1 +/− under Fe/DIP); rows 3 and 4, Pho7-3sAID strain (Pho7 +/− under Fe/DIP); and rows 5 and 6: Fep1-3sAID × Pho7-3sAID double mutant (Fep1 +/−, Pho7 +/− under Fe/DIP). Data represent the mean ± SD from three independent biological experiments. Statistical significance: ****P < 0.0001; ***P < 0.001; **P < 0.01; *P < 0.05; and ns, not significant. (**F**) Quantitative RT-PCR analysis of iron-depleted target genes (*pdc201+*, *tna1+*, and *pho84+*) upon depletion of Fep1, Pho7, or both factors under iron-replete and iron-depleted conditions. Rows 1 and 2, Fep1-3sAID strain (Fep1 +/− under Fe/DIP); rows 3 and 4, Pho7-3sAID strain (Pho7 +/− under Fe/DIP); and rows 5 and 6, Fep1-3sAID × Pho7-3sAID double mutant (Fep1 +/−, Pho7 +/− under Fe/DIP). Data represent the mean ± SD from three independent biological experiments. Statistical significance: ****P < 0.0001; ***P < 0.001; **P < 0.01; *P < 0.05; and ns, not significant.

We compared the two gene sets and found that Fep1 and Pho7 co-occupied a substantial number of promoters—specifically, they shared 178 genes between them under iron-replete conditions (Fig. 6A; Table S6A). Subsequently, we selected the previously uncharacterized genes *SPACUNK4.15*, *dps1+*, and *pdf1+* as representative targets from this group. IGV analysis confirmed that both Fep1-5FLAG and Pho7-13MYC exhibited increased binding at these promoters under iron-abundant condition (Fig. 6B).

Under iron-depleted conditions, we identified a distinct and smaller set of 25 co-occupied genes (Fig. 6C; Table S6B). These loci were characterized by increased Pho7-13MYC binding under iron-depleted conditions, which was consistent with the enhanced Pho7 recruitment observed under attenuation of Fep1 activity. From this set, *pdc201+* and *tna1+* were selected as novel targets along with the established Pho7 target *pho84+*. IGV analysis confirmed that binding of both Fep1-5FLAG and Pho7-13MYC at these promoters was preferentially elevated under iron-depleted conditions (Fig. 6D).

We determined the mechanisms underlying the Fep1–Pho7-mediated functional regulation of transcription at these shared targets by performing RT-qPCR analysis using Fep1-3sAID, Pho7-3sAID, and the double-degron strains after acute IAA treatment under iron-replete and iron-depleted conditions (Fig. 6E and F). With regard to genes co-occupied under iron-replete conditions (*SPACUNK4.15*, *dps1+*, and *pdf1+*), transcript levels were strongly induced upon Fep1 depletion regardless of iron status, whereas Pho7 depletion exerted minimal effects, which indicated that Fep1 acts as the dominant repressor at these loci (Fig. 6E). In contrast, at genes co-occupied under iron-depleted conditions (*pdc201+*, *tna1+*, and *pho84+*), the transcript levels were substantially reduced upon Pho7 depletion under both iron conditions, which establishes Pho7 as the primary activator (Fig. 6F). Fep1 depletion caused only modest increase in *tna1+* and *pho84+* expression under iron-replete conditions, which was consistent with a limited regulatory contribution by Fep1 at these targets.

Collectively, these analyses indicate that although Fep1 and Pho7 bind to several of the same promoters, the functional hierarchy between the two transcription factors is dictated by iron availability, with Fep1 predominating at targets co-occupied under iron-replete conditions and Pho7 acting as the dominant regulator at targets co-occupied under iron depletion.

## DISCUSSION

In this study, we implemented genome-wide ChIP-seq and RNA-seq approaches in combination with acute genetic perturbation to examine the manner in which the iron-responsive repressor Fep1 and phosphate-responsive activator Pho7 contribute to transcriptional regulation in *S. pombe*. The findings presented in this study expand the known target repertoires of both transcription factors and indicate an extensive overlap in their chromatin binding profiles across nutrient conditions. In addition to canonical iron uptake genes bound by Fep1 and phosphate-responsive genes regulated by Pho7, we have identified a substantial number of loci at which both factors associate with promoter regions. Unexpectedly, this included multiple phosphate-responsive genes and the *pho7*+ locus itself among Fep1-bound regions, which suggests that Fep1-mediated regulation extends beyond classical iron homeostasis pathways.

Acute depletion experiments using the AID system showed that Pho7 removal produced little global change in Fep1 chromatin association, whereas Fep1 loss resulted in a pronounced increase in Pho7 occupancy at a subset of loci, particularly among strongly iron-responsive promoters. This binding asymmetry implies that Fep1 can oppose Pho7 recruitment at specific targets, which is consistent with a model in which Fep1 activity directly or indirectly limits Pho7 access or stabilization at shared promoters. However, the molecular basis of this antagonism remains unresolved; hence, an important next step is to distinguish between (i) direct competition (e.g., overlapping or adjacent motifs that cannot be co-occupied), (ii) chromatin-mediated restriction (Fep1-dependent nucleosome positioning or histone modification states that disfavor Pho7 binding), and (iii) cofactor partitioning (shared co-regulators preferentially recruited by Fep1 under iron sufficiency). Future experiments, including promoter motif mutagenesis, reporter assays, chromatin accessibility or nucleosome-positioning analyses, and cofactor recruitment assays, will be required to distinguish among these possibilities and determine whether Fep1 limits Pho7 binding through direct promoter competition or indirect chromatin- or cofactor-dependent mechanisms.

Transcriptional analyses indicated that Pho7 contributes to the basal expression of a subset of Fep1 target genes under iron-limiting and weakened Fep1 repression conditions. Under iron-depleted conditions, Pho7 depletion reduced the transcript levels of representative iron uptake genes, such as *fio1*+/*fip1*+ and *str3*+, which was consistent with the action of Pho7 as an activator at these loci under iron starvation. Additionally, this functional contribution is supported by the strong growth defect observed after Pho7 depletion specifically under iron limitation, which suggests that Pho7-dependent transcription becomes particularly important for cellular fitness under iron scarcity. Overall, these results support a regulatory scenario in which Pho7 helps sustain the expression of select iron-uptake modules under low-iron conditions, whereas under iron-replete conditions the Fep1 repressive program predominates, and the presence of Pho7 exerts limited impact on those same iron targets.

**Fig 7:**
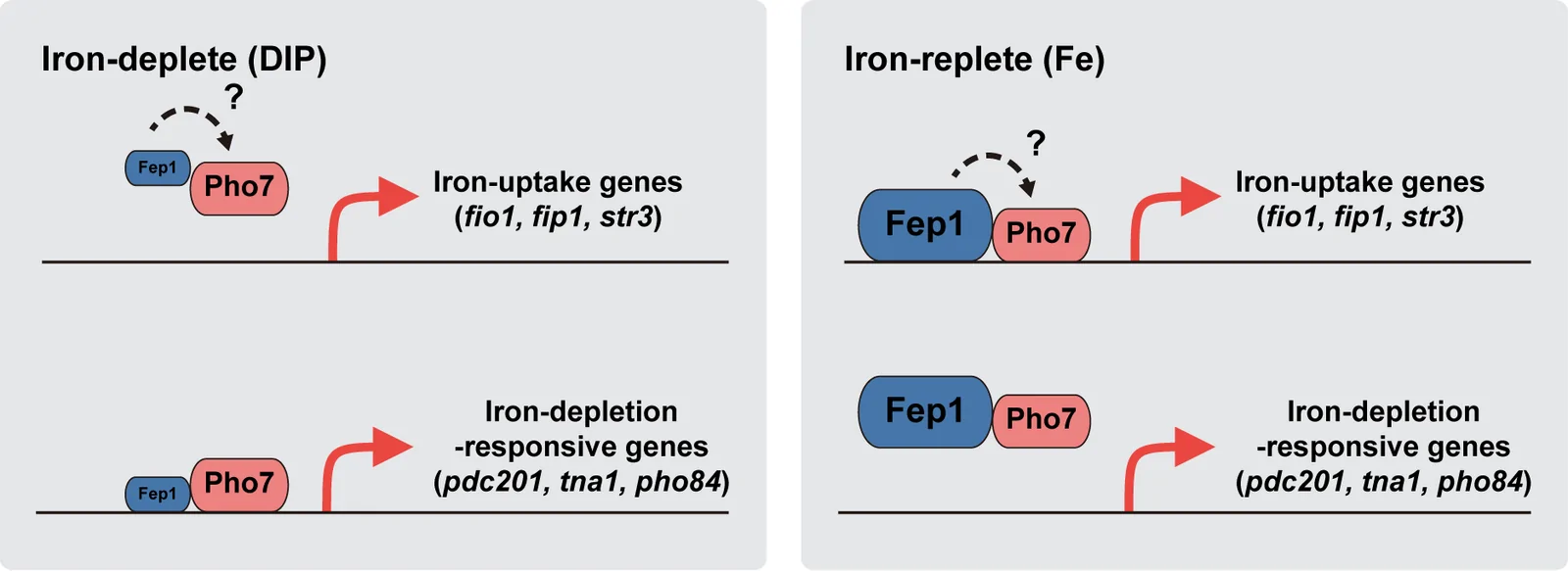
Iron-dependent cross-regulation of Fep1 and Pho7 at shared target promoters. Schematic model summarizing how iron availability redistributes the relative contributions of Fep1 and Pho7 to transcriptional control of iron-uptake genes (*fio1+*, *fip1+*, and *str3+*) and iron-depletion-responsive genes (*pdc201+*, *tna1+*, and *pho84+)*. Under iron-depleted conditions, Pho7 preferentially activates a subset of iron-uptake genes and iron-depletion-responsive targets, whereas under iron-replete (Fe) conditions, Fep1 becomes the dominant regulator at iron-uptake promoters while Pho7 retains a more limited activating role. The dashed arrows with question marks indicate regulatory steps in which the mechanistic basis of Fep1-Pho7 interplay remains unresolved; for specific details, please see Discussion.

To integrate these findings, we propose a working model in which iron availability dictates the hierarchical relationship between Fep1 and Pho7 at shared promoters (Fig. 7). Under iron-depleted conditions, both Fep1 and Pho7 preferentially associate with iron-depletion-responsive targets, such as *pdc201+*, *tna1*+, and *pho84+*, whereas iron-uptake genes, including *fio1+*, *fip1*+, and *str3*+ are largely unbound. When iron is abundant, this pattern is reversed: Fep1 and Pho7 co-occupy promoters of iron-uptake genes, while their binding to iron-depletion-responsive genes is reduced. However, because intracellular iron and phosphate pools were not quantitatively measured in this study, we cannot exclude the possibility that some of the observed transcriptional changes reflect indirect metabolic coupling rather than direct transcriptional cross-regulation by Fep1 and Pho7. Within this framework, acute Fep1 depletion increases Pho7 occupancy at a subset of shared targets, particularly at iron-uptake genes, suggesting that Fep1 normally limits Pho7 association with these promoters. Whether this restriction occurs through direct competition, changes in the local chromatin environment, or altered cofactor usage remains unresolved and is therefore represented as an undefined step in our model.

The unexpected association of Fep1 with iron-depletion-responsive promoters under iron-limiting conditions also raises questions regarding the DNA-recognition properties of its iron-depleted form. *De novo* motif analysis of promoters showing clear Fep1-5FLAG enrichment identified three enriched sequence motifs, 5′-AGCTCT-3′, 5′-AATTGGTMK-3′, and 5′-GCCTAT-3′, none of which correspond closely to the canonical Fep1-binding motif associated with iron-dependent repression. These preliminary observations raise the possibility that apo-Fep1 may associate with a distinct set of cis-regulatory elements when iron is limiting. However, the present data do not establish whether apo-Fep1 directly recognizes these motifs or whether its promoter association is mediated or stabilized by additional DNA-binding proteins or transcriptional cofactors. It also remains unclear whether promoter-bound apo-Fep1 retains an active regulatory function or represents a chromatin-associated state with limited transcriptional activity. Therefore, we interpret the identified motifs as candidates for further investigation rather than as definitive apo-Fep1 response elements. Targeted motif mutagenesis, promoter-reporter analyses, identification of Fep1-associated cofactors, and direct biochemical characterization of apo-Fep1–DNA interactions will be required to determine whether iron depletion alters the DNA-recognition mode of Fep1 or promotes its recruitment through cofactor-dependent mechanisms.

In a broad sense, these results support the expansion of nutrient cross-regulation beyond iron–phosphate to encompass a wider multi-element homeostasis network in fission yeast. In plants, phosphate–iron–zinc–sulfur interactions are being increasingly recognized as integrated systems that are coordinated by shared regulators and signaling nodes (16, 17). Although the molecular wiring differs in fungi, *S. pombe* possesses well-defined transcriptional regulators for other elemental stresses, including the zinc-responsive transcription factor Loz1, which binds to specific response elements and represses gene expression in zinc-replete conditions. Hence, future studies must extend the scope of the experimental framework used here to examine the interactions between iron, phosphate, zinc, and sulfur regulatory systems. Particularly, the line of investigation would help determine whether the coordination observed between Fep1 and Pho7 reflects a general strategy for integrating elemental signals or a more specialized regulatory relationship.

In summary, the results of the present study define a transcriptional framework in which Fep1 and Pho7 share a subset of target genes but contribute to their regulation in a nutrient-dependent manner. Under iron sufficiency, Fep1-mediated repression predominates, whereas under iron limitation, Pho7 supports basal transcription of selected iron uptake genes. These findings establish a foundation for dissecting the mechanisms underlying the cooperation between nutrient-responsive transcription factors that maintain elemental homeostasis in fission yeast.

## MATERIALS AND METHODS

### *S. pombe* strains and cell culture

All *S. pombe* strains used in this study are listed in Table S1. The wild-type strain SPK425 was used as the parental background. Strains FY39923 and FY39926, which express *Padh1-OsTIR1-F74A*, were obtained from the National Bio-Resource Project (Japan Agency for Medical Research and Development, Japan). Epitope tags (Flag and Myc) and fluorescent protein tags (GFP and mCherry) were integrated at the C-terminus of target genes using a PCR-based module method. For auxin-inducible degradation systems, the auxin-inducible degron peptide 5-Ada-IAA was integrated into strains FY39923 and FY39926 as described previously (42).

All strains were cultured in Edinburgh Minimal Medium at 30°C. To deplete proteins via the auxin-inducible degradation system, mid-log phase cells (OD_600_ = 0.5) were treated with 100 nM 5-adamantyl-IAA (TCI, A3390) for 1 h. For iron repletion, mid-log phase cells were supplemented with 100 µM ferric chloride (Sigma, F2877), and iron repletion was achieved by adding 250 µM 2,2′-bipyridyl (Sigma, D216305) for 1.5 h at 30°C. To emulate phosphate repletion and starvation conditions, mid-log phase cells grown in EMM medium were transferred to fresh EMM medium (phosphate-replete, +P) or EMM-P medium (phosphate-starved, −P) and cultured for 4 h at 30°C.

### ChIP-seq and data analysis

Chromatin immunoprecipitation was performed as previously described (43) with modifications, which can be found in the Supplemental material.

### RNA extraction and real-time quantitative PCR

RNA was purified from cells using the RNeasy Mini Kit (QIAGEN, 74104) by following the manufacturer’s instructions. The procedure is explained in detail in the Supplemental material.

### RNA-seq and data analysis

Messenger RNA was isolated to prepare RNA-seq libraries. The reads were aligned to the *S. pombe* genome, and gene expression was quantified by determining the transcripts per million values followed by analysis of differentially expressed genes. These procedures are explained in detail in the Supplemental material.

### Western blotting

The procedures followed for western blotting, including the antibodies used, are explained in detail in the Supplemental material.

## Data Availability

The raw sequencing reads generated in this study have been deposited in the NCBI Gene Expression Omnibus (GEO) under accession number GSE344572.

## SUPPLEMENTAL MATERIAL

Supplemental materials spectrum.00716-26-s0001.pdf Supplemental methods and Figures S1 to S7.

Table S1 spectrum.00716-26-s0002.xlsx List of *S. pombe* strains used in this study.

Table S2 spectrum.00716-26-s0003.xlsx Genes showing increased Fep1-5FLAG binding under iron-replete versus iron-depleted conditions (FC ≥ 1.5; *n* = 270).

Table S3 spectrum.00716-26-s0004.xlsx Integrated Pho7 target genes upregulated upon Pho7-3sAID-mediated depletion (*n* = 22).

Table S4 spectrum.00716-26-s0005.xlsx Genes exhibiting enriched Fep1 and Pho7 chromatin occupancy in reciprocal AID strains (RPKM ≥ 300; *n* = 270).

Table S5 spectrum.00716-26-s0006.xlsx Genes co-occupied by Fep1-5FLAG and Pho7-13MYC under iron-depleted conditions (RPKM ≥ 300; *n* = 264).

Table S6 spectrum.00716-26-s0007.xlsx Genes co-enriched for Fep1-5FLAG and Pho7-13MYC binding under iron-depleted conditions (FC ≥ 1.5; *n* = 25).

ASM does not own the copyrights to Supplemental Material that may be linked to, or accessed through, an article. The authors have granted ASM a non-exclusive, world-wide license to publish the Supplemental Material files. Please contact the corresponding author directly for reuse.
