# Supplementary material for: Cross-regulation of iron and phosphate homeostasis by Fep1 and Pho7 in fission yeast
Source: Microbiol Spectr. 2026 Oct 2;14(11):e00716-26. doi: 10.1128/spectrum.00716-26 (PMC13729207; doi:10.1128/spectrum.00716-26)
Supplement: Supplementary file 1 [file spectrum.00716-26-s0001.pdf]

Supplemental Materials

Supplemental Methods

Supplemental Figures 1-7

Supplemental Table 1-6

Supplemental Methods

ChIP-seq and Data Analysis

The cells were cross-linked with 3% paraformaldehyde at 18°C for 30 min, followed by secondary cross-linking with 10 mM dimethyl adipimidate at 25°C for 45 min. Cell lysis was performed by bead beating in ChIP lysis buffer (50 mM HEPES-KOH [pH 7.5]; 140 mM NaCl; 1 mM EDTA [pH 8.0]; 1% Triton X-100; 0.1% sodium deoxycholate; and 0.1 mM PMSF) supplemented with protease inhibitors (cOmplete, Roche, 11836153001). The lysates were fragmented through sonication with a Bioruptor (Diagenode), and pre-clearing was performed with protein G Dynabeads (Thermo, 10004D) at 4°C for 1 h. Antibodies against either anti-DDDDK-tag (MBL, M1853L) or anti-Myc (MBL, M047-3) were pre-incubated with fresh Dyna-G beads for ≥ 4 h at 4°C to prepare antibody-bound beads. Immunoprecipitation was performed by incubating the sonicated lysates with the antibody-bound beads overnight at 4°C. The beads were washed sequentially with ChIP lysis buffer (twice), lysis buffer containing 650 mM NaCl, ChIP wash buffer (10 mM Tris-HCl [pH 8.0]; 250 mM LiCl; 0.5% NP-40; 0.5% sodium deoxycholate; and 1 mM EDTA), and TE buffer. DNA was eluted by incubating beads in TE buffer containing 1% SDS at 65°C for 15 min with shaking (1000 rpm). The elution was repeated twice. Next, the samples were incubated overnight at 65°C, followed by treatment with proteinase K at 37°C for 5 h. Then, ChIP DNA was purified using the QIAquick PCR Purification Kit (QIAGEN, 28104).

ChIP-seq libraries were prepared using the NEBNext Ultra II DNA Library Prep Kit for Illumina (NEB, E7645) and NEBNext Multiplex Oligos for Illumina (NEB, E7335, Index Primers Set 1) with amplification using Q5 Master Mix (NEB, M0544). The DNA fragments that were 200–700 bp were enriched using AMPure XP beads (Beckman Coulter, A63881) and subjected to 100 bp paired sequencing on an Illumina NovaSeq platform. The reads were

aligned to the *S. pombe* genome using Bowtie2 (v2.2.5), and peaks were called and visualized in TDF format using IGV tools. To quantify ChIP enrichment, the reads per kilobase million (RPKM) values were determined within a 500–bp region surrounding the transcription start site (TSS).

RNA Extraction and Real-Time Quantitative PCR (RT-qPCR)

Contaminating genomic DNA was eliminated by treatment with the TURBO DNA-free kit (Thermo, AM1907). Next, RNA concentration and purity were measured using a Qubit fluorometer (Thermo, Q33226). Complementary DNA (cDNA) was synthesized from 1 μg of total RNA using RevertAid Reverse Transcriptase (Thermo, EP0442) with random hexamer primers. Relative transcript abundance was determined via real-time quantitative PCR using HOT FIREPol EvaGreen qPCR Mix Plus (Solis BioDyne, 08-24-00001) with gene-specific primers. The expression levels were calculated using ΔΔCt method with *act1*+ as the reference gene.

RNA-seq and Data Analysis

Messenger RNA was isolated from 1 μg of total RNA using Poly(A) mRNA Magnetic Isolation (NEB, E7490) according to the manufacturer's instructions. RNA-seq libraries were constructed using the NEBNext Ultra RNA Library Prep Kit for Illumina (NEB, E7770) and NEBNext Multiplex Oligos for Illumina (NEB, E7335, Index Primers Set 1) with amplification using Q5 Master Mix (NEB, M0544). cDNA synthesis and conversion to double-strand DNA were performed as part of the library preparation workflow. DNA fragments in the 200–700 bp range were enriched using AMPure XP beads (Beckman Coulter, A63881) and sequenced as

100–bp paired end reads on an Illumina NovaSeq platform. The reads were aligned to the *S. pombe* genome using Bowtie2 (v2.2.5). Gene expression was quantified by determining the transcripts per million (TPM) values. Analysis of differentially expressed genes were performed using the DESeq2 R package (version 1.44.0)

Western Blotting

Mid-log phase cells were lysed in 0.1 M potassium phosphate buffer supplemented with protease inhibitors and 1 mM PMSF using bead beating for cell disruption. Crude lysates were collected through centrifugation, and protein concentrations were determined using the Qubit Protein Assay Kit (Thermo, A50669). The samples were normalized for total protein content, mixed with 4X Laemmli sample buffer, and boiled for 5 min. Equal quantities of proteins were resolved on 8% SDS-PAGE gels and transferred onto nitrocellulose membranes (GE Healthcare, 10600004), which were incubated with Smat-Block™ Fast Blocking Buffer (Biomax, BWB-0500) at 25°C for 10 min to block nonspecific binding. Overnight incubation was performed at 4°C with primary antibodies targeting mini-AID (1:500, MBL, M214-3), Myc (1:2500, MBL, M047-3), DDDDK-tag (1:5000, MBL, M185-3L), and β-tubulin (1:5000, BioAcademia, 63-160). After six TBST washes, secondary antibody detection was performed at room temperature for 1 h using goat anti-mouse IgG HRP-conjugated antibody (Bio-Rad, 170-6516) for the tagged proteins and goat anti-rabbit IgG HRP-conjugated antibody (Bio-Rad, 170-6515) for the *S. pombe*-specific rabbit polyclonal anti-β-tubulin antibody. Chemiluminescent detection was performed using WestGlow™ ECL reagents (Biomax, BWE0400), and the images were captured on an e-blot imager. Band intensities were quantified using ImageJ software for densitometric analysis.

De novo Motif Discovery Using MEME-ChIP

Promoter sequences located 1 kb upstream of the annotated TSS were extracted from the *S. pombe* reference genome that was used for mapping the ChIP-seq and RNA-seq reads with Bowtie2 (v2.2.5). The resulting FASTA file was analyzed with MEM-ChIP (MEM suite v5.5.9) via the MEME Suite web server. For motif discovery, STREAM and MEME were run with a second-order Markov background model derived from the input sequences, and both DNA strands were scanned. MEME was configured in ZOOPS mode ("zero or one occurrence per sequence") to discover up to three motifs with widths between 6 and 15 bp. At least two sites per motif were required, and any number of motif repetitions per sequence were allowed. STREAM was run at default setting with the exception of the minimum and maximum motif widths being set to 6 and 15 bp, respectively, and up to five motifs being requested. The motifs with STREAM *P* values ≤ 0.05 were retained for further analysis.

Supplemental Figures

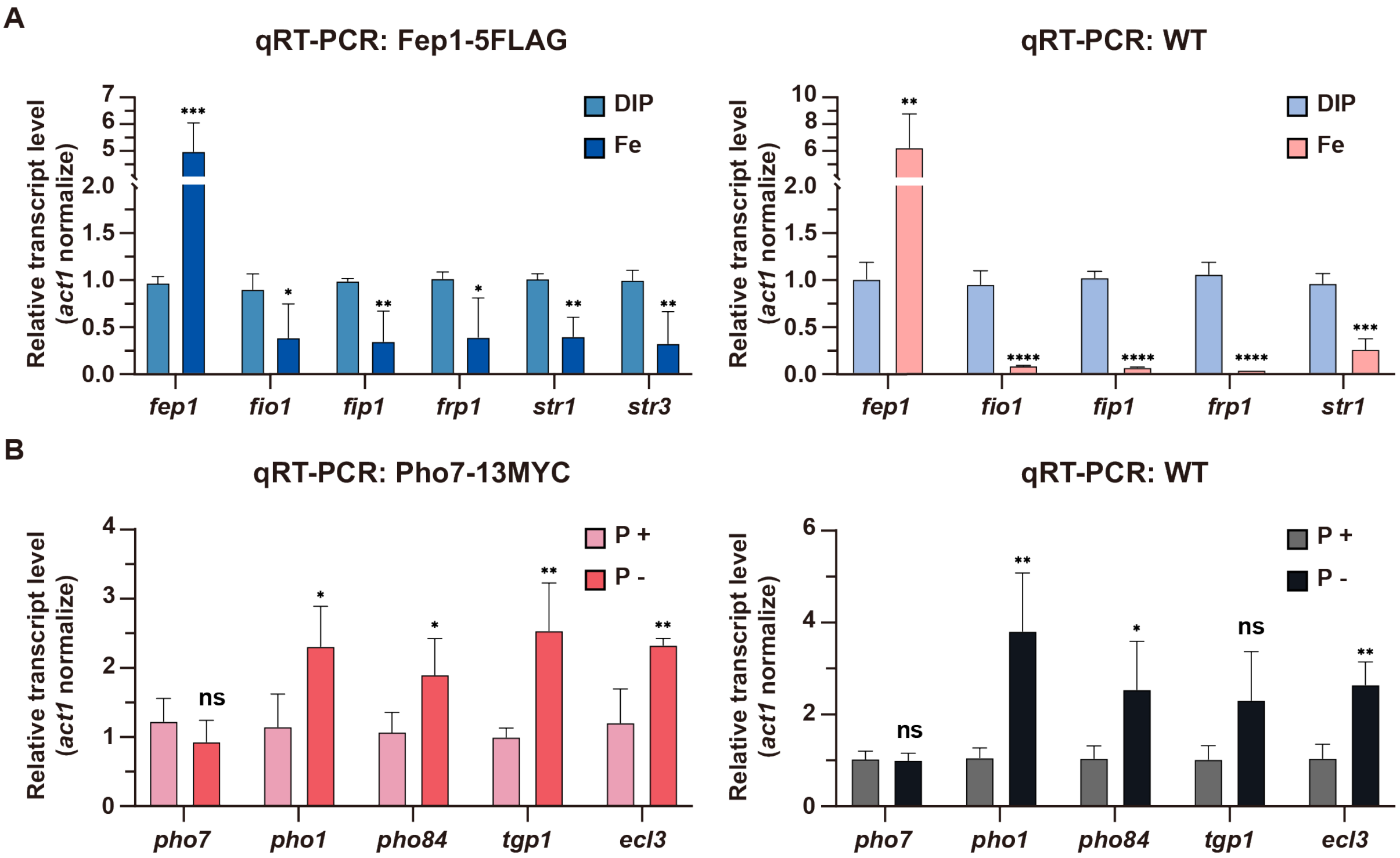

Figure S1. RT-qPCR analysis of Fep1 target genes in Fep1-5FLAG and wild-type cells under iron-modulated conditions and Pho7 target genes in Pho7-13MYC and wild-type cells under phosphate modulated conditions.

(A) Quantitative RT-PCR analysis of Fep1 target genes (iron-uptake genes: *fio1+, fip1+, frp1+, str1+, str3+*) in the Fep1-5FLAG and wild-type (WT) strains grown under iron-replete conditions (100 μM $FeCl_3$, 1.5h) or iron depleted conditions (250 μM DIP, 1.5h). Transcript levels were normalized to *act1+*. Statistical significance: ****$P < 0.0001$; ***$P < 0.001$; **$P < 0.01$; *$P < 0.05$; *ns* (not significant).

(B) Quantitative RT-PCR analysis of Pho7 target genes (PHO pathway genes: *pho1+, pho84+, tgp1+* and *ecl3+*) in the Pho7-13MYC and WT strains grown under phosphate-replete (P+) or phosphate-starved (P-; 4h in EMM-P) conditions. Transcript levels were normalized to *act1+*. Statistical significance: ****$P < 0.0001$; ***$P < 0.001$; **$P < 0.01$; *$P < 0.05$; *ns* (not significant).

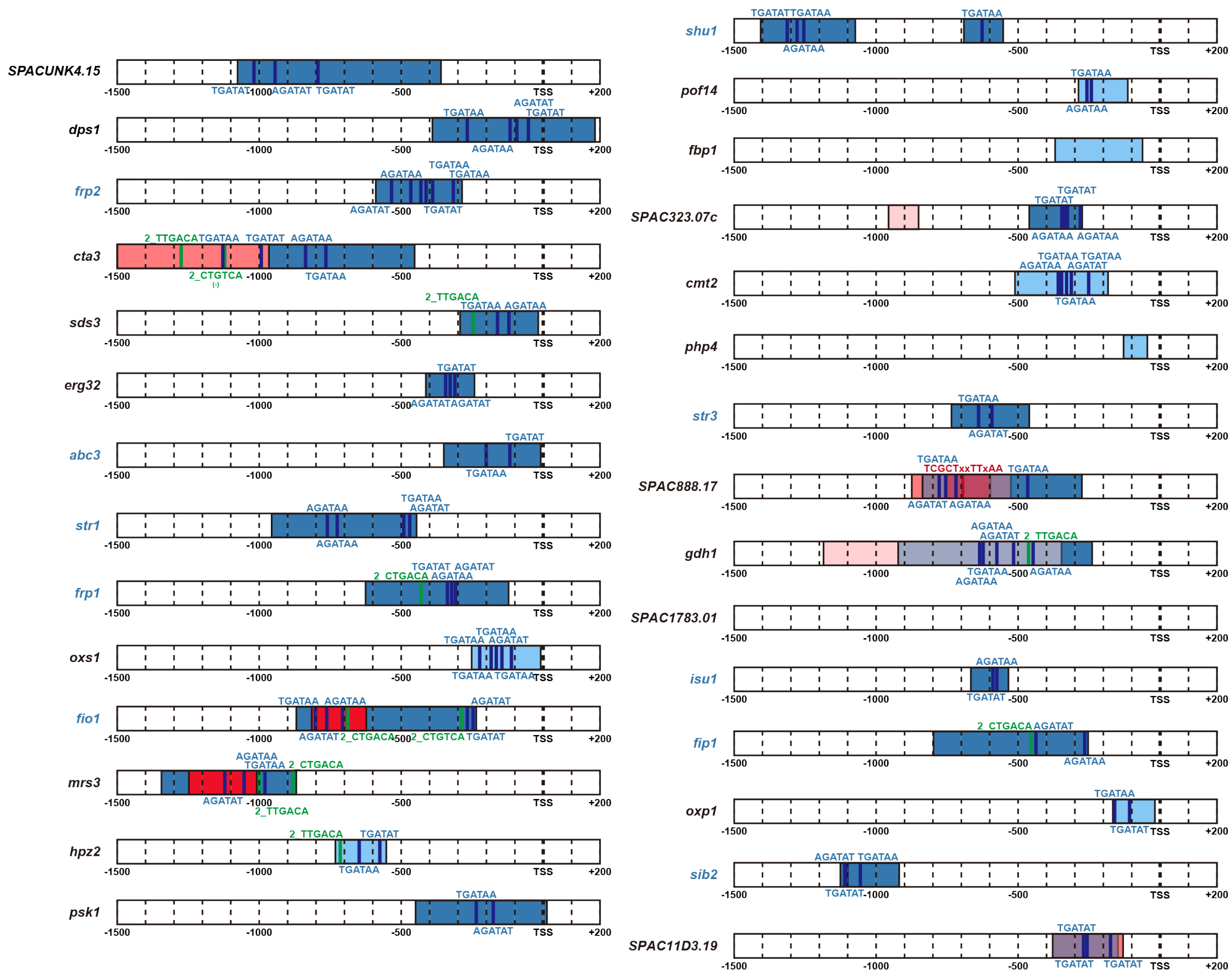

Figure S2. Fep1 binding motifs and ChIP-seq enrichment profiles at promoters of Fep1 target genes.

Schematic representation of Fep1-5FLAG ChIP-seq binding profiles and Fep1 consensus motifs in the promoters of the Fep1 target genes identified in Figure 1F (n=29). For each gene, the region from -1500 to +200 nt relative to the transcription start site (TSS) is shown. Blue bars indicate Fep1 binding enrichment detected by Fep1-5FLAG ChIP-seq under iron-depleted (DIP) or iron-replete (Fe) conditions; dark blue denotes regions with normalized read counts ≥4000, and light blue denotes regions with normalized read counts ≥2000. Red bars indicate Pho7 binding enrichment detected by Pho7-13MYC ChIP-seq; Red denotes regions with normalized read counts ≥4000, light red denotes regions with normalized read counts ≥2000.

The Fep1 consensus binding motif (A/T)GATA(A/T) is annotated in blue, the Pho7 consensus binding motif TCG(G/C)(A/T)xxTTxAA is annotated in red, and the two Pho7 motifs (CWTCTTAA, YTGACA) identified in Figure S4B are annotated in green, each with their position relative to the TSS. Tick marks on the x-axis indicate nucleotide distance relative to the TSS. Detailed motif sequences, their orientations, and genomic coordinates are provided in the corresponding Table S2D.

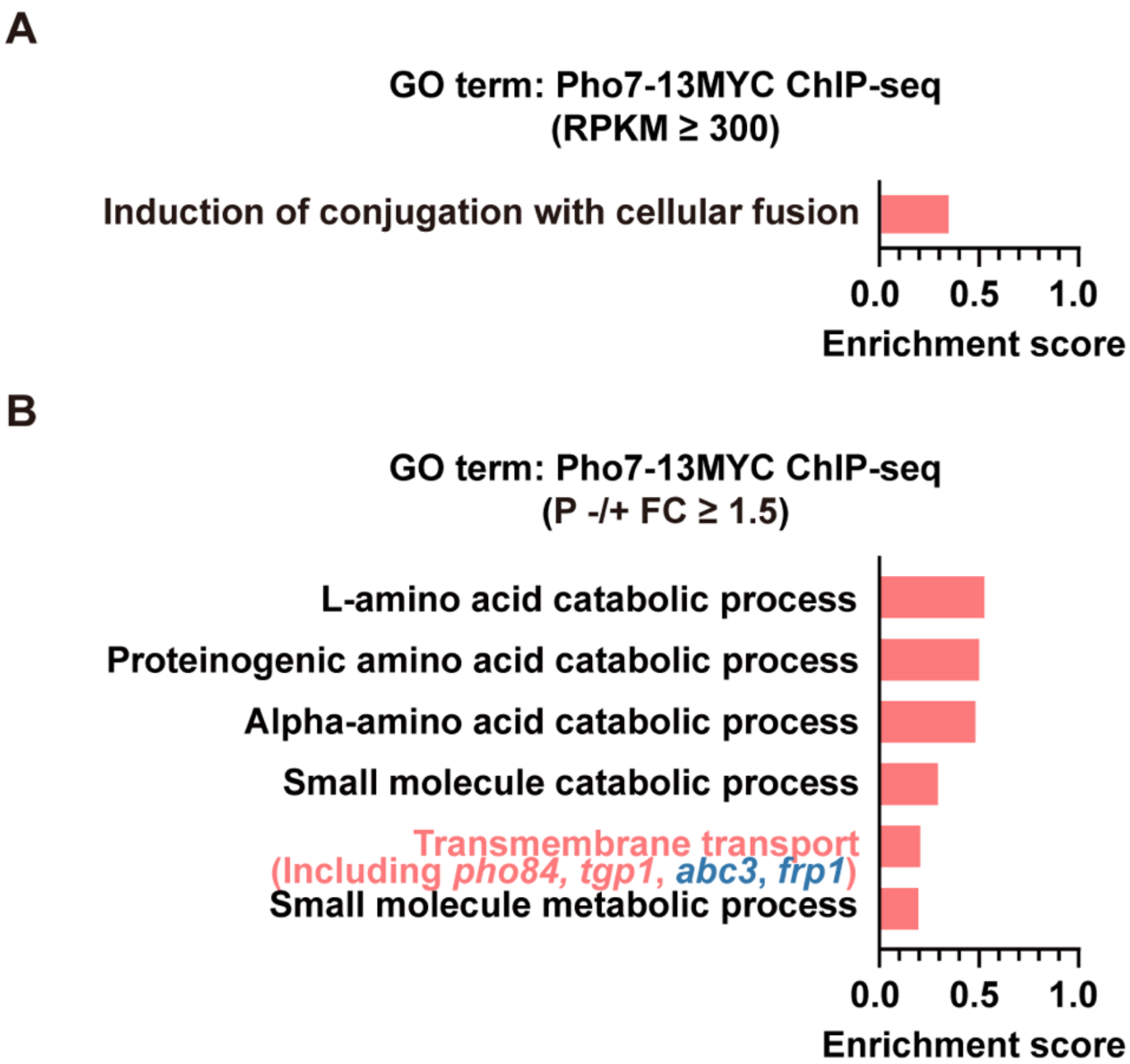

Figure S3. Gene Ontology enrichment analysis of Pho7-13MYC ChIP-seq targets.

(**A**) GO enrichment analysis of the 270 Pho7-bound genes identified in Pho7-13MYC ChIP-seq (RPKM ≥300). The most significantly enriched biological process term is displayed.

(**B**) GO enrichment analysis of 584 Pho7-bound genes with ≥1.5-fold increased Pho7-13MYC ChIP-seq binding under phosphate starvation relative to phosphate-replete conditions (-P/+P; Fig. 2D). The top enriched biological process terms are shown, ranked by enrichment score.

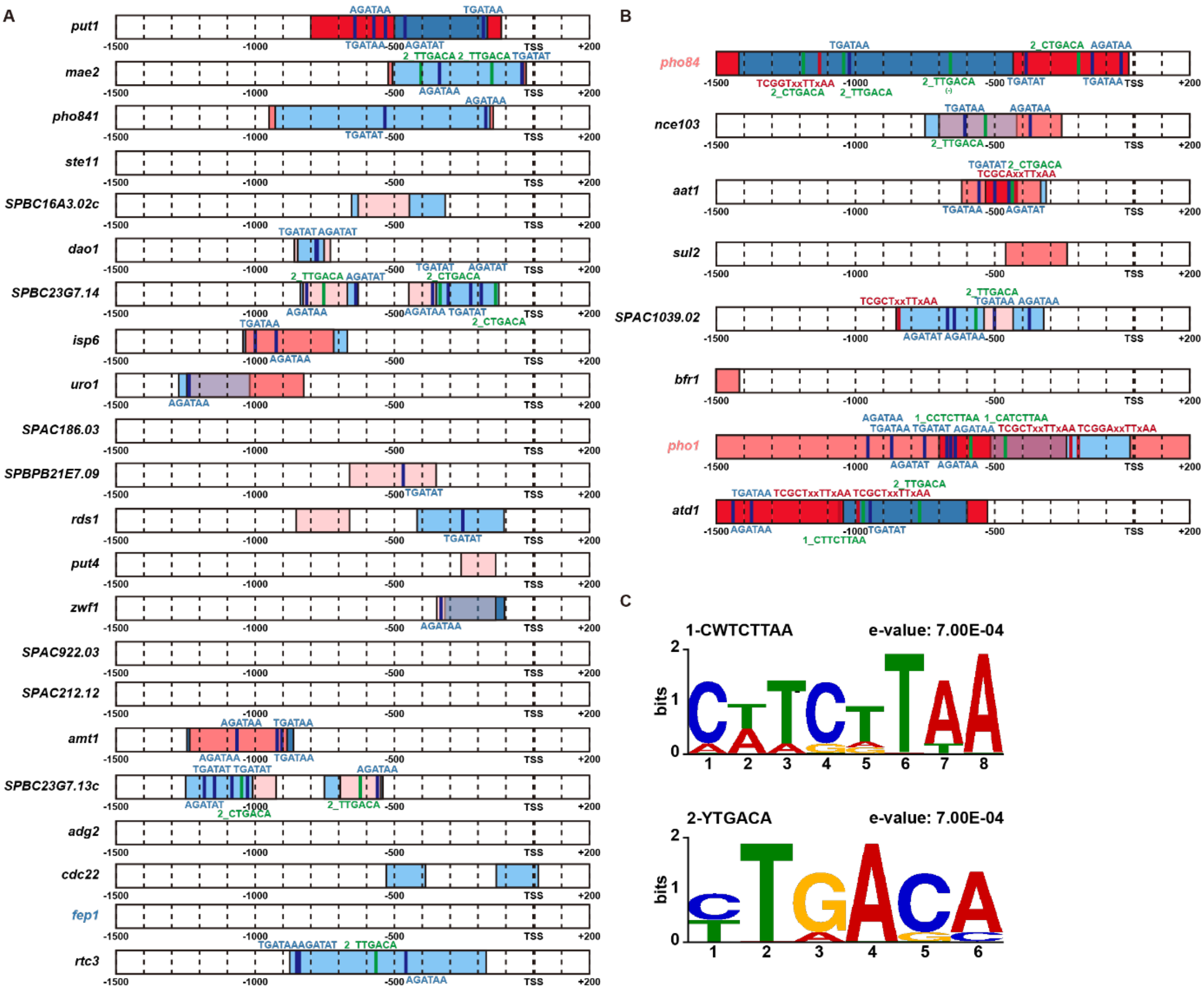

Figure S4. Pho7 binding motifs and ChIP-seq enrichment profiles at promoters of Fep1 target genes.

(A) Schematic representation of Pho7-13MYC ChIP-seq binding profiles and Pho7 consensus motifs in the promoters of the Pho7 target genes identified in Figure 2F (Upregulation, n=22). For each gene, the region from -1500 to +200 nt relative to the transcription start site (TSS) is shown. Red bars indicate Pho7 binding enrichment detected by Pho7-13MYC ChIP-seq under phosphate-replete (P+) or P-depleted (P-) conditions; Red denotes regions with normalized read counts ≥4000, light red denotes regions with normalized read counts ≥2000, and pink denotes regions with normalized read counts ≥1500. Blue bars indicate Fep1 binding enrichment detected by Fep1-5FLAG ChIP-seq; dark blue denotes regions with normalized

read counts ≥ 4000, and light blue denotes regions with normalized read counts ≥ 2000. The Fep1 consensus binding motif (A/T)GATA(A/T) is annotated in blue, the Pho7 consensus binding motif TCG(G/C)(A/T)xxTTxAA is annotated in red, and the two Pho7 motifs (CWTCTTAA, YTGACA) identified in Figure S3C are annotated in green, each with their position relative to the TSS. Tick marks on the x-axis indicate nucleotide distance relative to the TSS. Detailed motif sequences, their orientations, and genomic coordinates are provided in the corresponding Table S3F.

(B) Schematic representation of Pho7-13MYC ChIP-seq binding profiles and Pho7 consensus motifs in the promoters of the Fep1 target genes identified in Figure 2F (Downregulation, n=8). For each gene, the region from -1500 to +200 nt relative to the transcription start site (TSS) is shown. Red bars indicate Pho7 binding enrichment detected by Pho7-13MYC ChIP-seq under phosphate-replete (P+) or P-depleted (P-) conditions; Red denotes regions with normalized read counts ≥ 4000, light red denotes regions with normalized read counts ≥ 2000, and pink denotes regions with normalized read counts ≥ 1500. The Fep1 consensus binding motif (A/T)GATA(A/T) is annotated in blue, the Pho7 consensus binding motif TCG(G/C)(A/T)xxTTxAA is annotated in red, and the two Pho7 motifs (CWTCTTAA, YTGACA) identified in Figure S3C are annotated in green, each with their position relative to the TSS. Tick marks on the x-axis indicate nucleotide distance relative to the TSS. Detailed motif sequences, their orientations, and genomic coordinates are provided in the corresponding Table S3G.

(C) De novo motif discovery from Pho7-depedent target promoters. Sequence logos of two candidate Pho7 recognition motifs identified by MEME-ChIP/STREAM analysis of the promoters of eight Pho7-dependnet genes: (top) motif 1-CWTCTTAA and (bottom) motif 2-YTGACA.

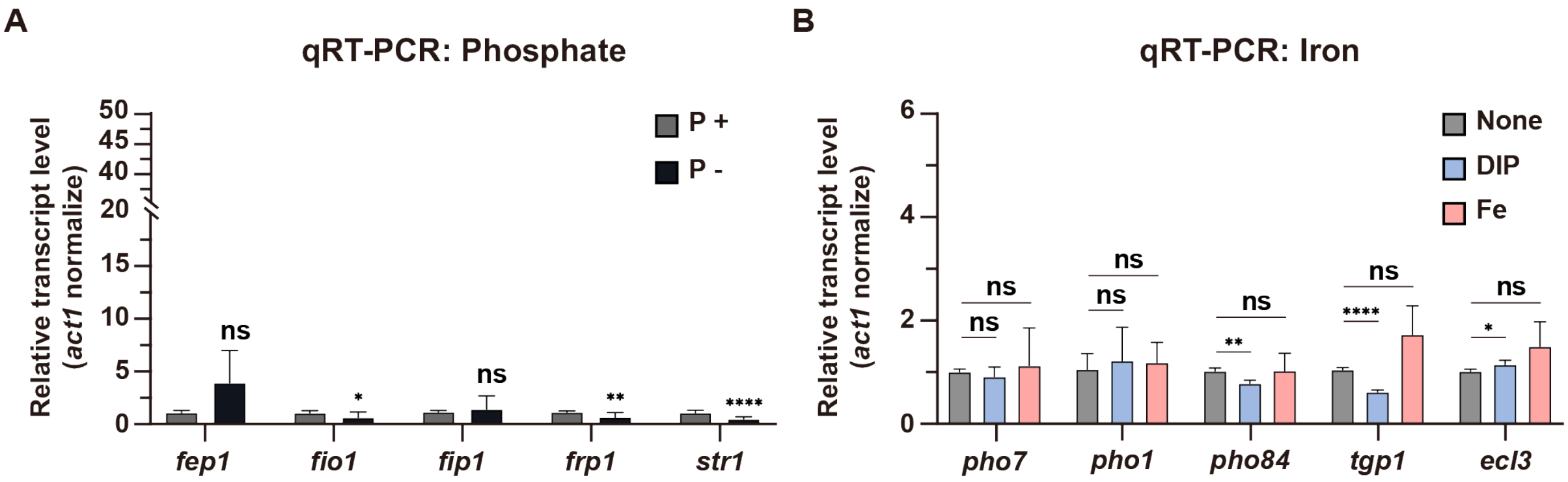

Figure S5. RT-qPCR analysis of Fep1 and Pho7 target genes under iron- and phosphate-modulated conditions.

(A) Quantitative RT-PCR analysis of Fep1 target genes (iron-uptake genes: *fio1+, fip1+, frp1+, str1+, str3+*) under phosphate-replete (P+) or phosphate-starved (P-; 4h in EMM-P) conditions. Transcript levels were normalized to *act1+*. Statistical significance: *****P* < 0.0001; ****P* < 0.001; ***P* < 0.01; **P* < 0.05; *ns* (not significant).

(B) Quantitative RT-PCR analysis of Pho7 target genes (PHO pathway genes: *pho1+, pho84+, tgp1+* and *ecl3+*) under iron-replete conditions (100 µM $FeCl_3$, 1.5h) or iron depletion conditions (250 µM DIP, 1.5h). Transcript levels were normalized to *act1+*. Statistical significance: *****P* < 0.0001; ****P* < 0.001; ***P* < 0.01; **P* < 0.05; *ns* (not significant).

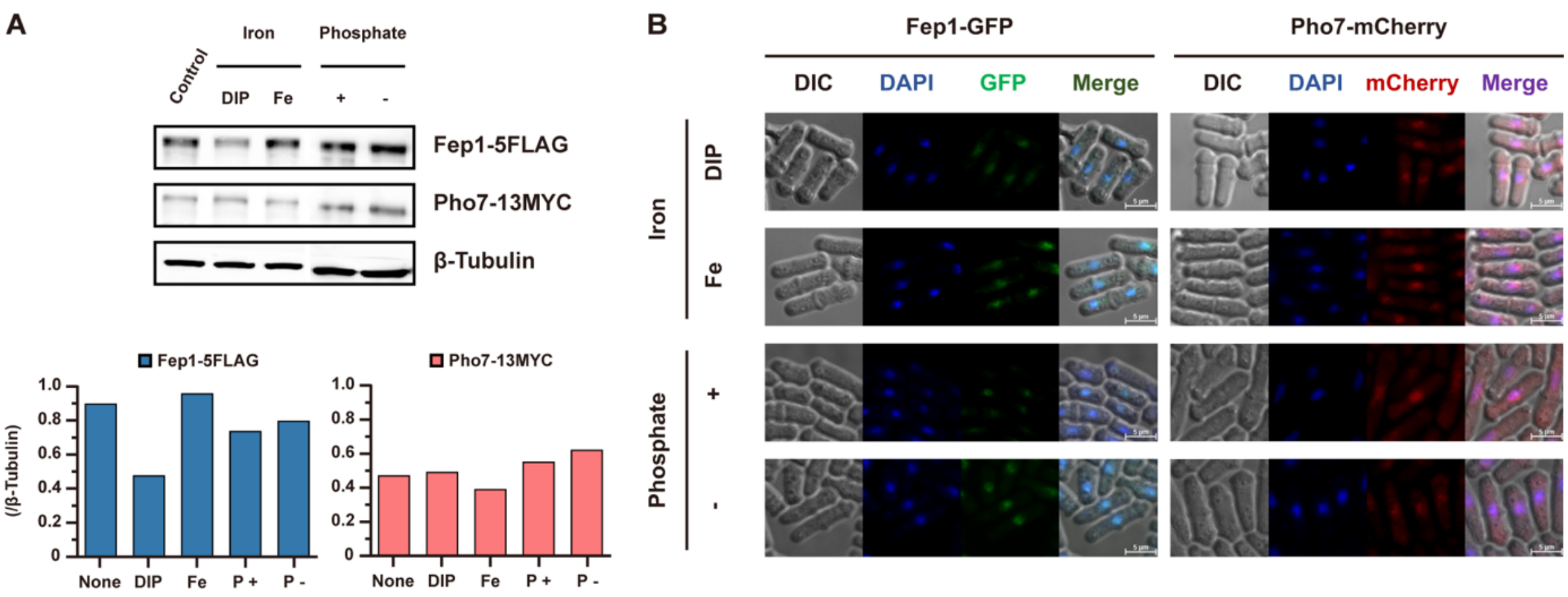

Figure S6. Fep1-5FLAG and Pho7-13MYC protein expression under iron- and phosphate-modulated conditions.

(A) Western blot analysis of Fep1-5FLAG and Pho7-13MYC protein levels under iron-depleted (DIP), iron-replete (Fe), phosphate-replete (P+), or phosphate-starved (P-) conditions. Representative blots (upper) and ImageJ quantification (lower) are shown after normalization to tubulin. Pho7-13MYC levels remain stable across all conditions, whereas Fep1-5FLAG levels increase under iron-replete conditions and decrease under iron-depleted conditions.

(B) Fluorescence microscopy images of Fep1-GFP (left) and Pho7-mCherry (right) localization under four nutrient conditions: iron-depleted (DIP), iron-replete (Fe), phosphate-replete (P+), and phosphate-starved (P-). Each row displays, differential interference contrast (DIC), DAPI-stained nuclei (blue), fluorescent protein signal (GFP or mCherry), and merged image. Colocalization of DAPI with Fep1-GFP and Pho7-mCherry signals in all conditions indicates that both transcription factors localize exclusively to the nucleus, independent of iron or phosphate availability. Scale bar, 5 μm.

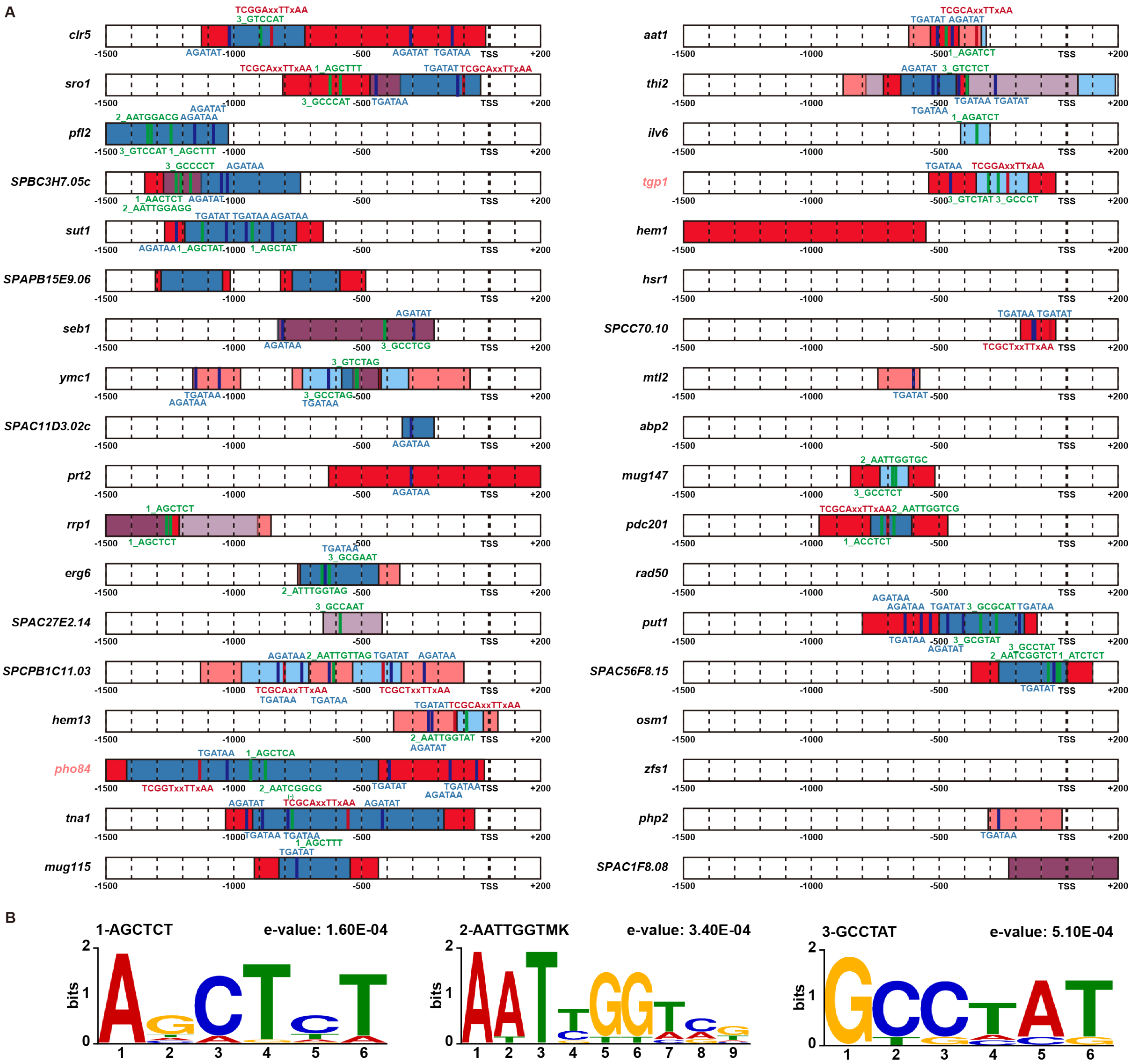

Figure S7. Fep1 binding motifs and ChIP-seq enrichment profiles at promoters of iron-depletion-responsive Fep1 targets

(A) Schematic representation of Fep1-5FLAG ChIP-seq binding profiles and Fep1 consensus motifs in the promoters of the Fep1 target genes identified in Figure 5D (n=36). For each gene, the region from -1500 to +200 nt relative to the transcription start site (TSS) is shown. Blue bars indicate Fep1 binding enrichment detected by Fep1-5FLAG ChIP-seq under iron-depleted (DIP) or iron-replete (Fe) conditions; dark blue denotes regions with normalized read counts ≥4000, and light blue denotes regions with normalized read counts ≥2000. Red bars indicate

Pho7 binding enrichment detected by Pho7-13MYC ChIP-seq; Red denotes regions with normalized read counts ≥4000, light red denotes regions with normalized read counts ≥2000. The three Fep1 motifs (AGCTCT, AATTGGTMK, GCCTAT) identified in Figure S7B are annotated in green, each with their position relative to the TSS. Tick marks on the x-axis indicate nucleotide distance relative to the TSS. Detailed motif sequences, their orientations, and genomic coordinates are provided in the corresponding Table S5F.

(B) De novo motif discovery from iron-depletion-responsive Fep1 target genes. Among 36 genes that showed increased Fep1-5FLAG enrichment under iron-depleted compared with iron-replete conditions. (Figure S7A), 25 genes with clear promoter Fep1-5FLAG enrichment peaks (blue bars) were selected and their promoter regions were subjected to MEME-ChIP/STREAM analysis. Sequence logos of three candidate Fep1 recognition motifs identified from these promoters are shown: (top) motif 1-AGCTCT, (middle) motif 2-AATTGGTMK, and (bottom) motif 3-GCCTAT.

Supplemental Tables

Table S1. List of *S. pombe* strains used in this study.

Table S2. Identification and functional analysis of Fep1 target genes.

Table S2A. Genes showing increased Fep1-5FLAG binding under iron-replete versus iron-depleted conditions (FC ≥ 1.5; n = 270).

Table S2B. GO term enrichment analysis of iron-responsive Fep1-5FLAG ChIP-seq targets (FC ≥ 1.5).

Table S2C. Genes upregulated upon Fep1-3sAID–mediated depletion (n = 32).

Table S2D. Fep1 target genes identified by integrated ChIP-seq and RNA-seq analysis (n = 29).

Table S2E. Genes with basal Fep1-5FLAG ChIP-seq occupancy (RPKM 300; n = 482).

Table S2F. GO term enrichment analysis of genes with basal Fep1-5FLAG occupancy (RPKM ≥ 300).

Table S3. Identification and functional analysis of Pho7 target genes.

Table S3A. Genes showing increased Pho7-13MYC binding under phosphate-depleted versus phosphate-replete conditions (FC ≥ 1.5; n = 584).

Table S3B. GO term enrichment analysis of genes with basal Pho7-13MYC occupancy (RPKM ≥ 300).

Table S3C. GO term enrichment analysis of phosphate-responsive Pho7-13MYC ChIP-seq targets (FC ≥ 1.5).

Table S3D. Genes upregulated upon Pho7-3sAID–mediated depletion (n = 39).

Table S3E. Genes downregulated upon Pho7-3sAID–mediated depletion (n = 11).

Table S3F. Integrated Pho7 target genes upregulated upon Pho7-3sAID–mediated depletion (n = 22).

Table S3G. Integrated Pho7 target genes downregulated upon Pho7-3sAID–mediated depletion (n = 8).

Table S4. Overlap between Fep1 and Pho7 regulatory targets.

Table S4A. Genes exhibiting enriched Fep1 and Pho7 chromatin occupancy in reciprocal AID strains (RPKM ≥ 300; n = 270).

Table S4B. Genes showing increased Pho7-13MYC binding upon Fep1 depletion (FC ≥ 1.5; n = 28).

Table S5. Condition-dependent co-occupancy of Fep1 and Pho7 at target promoters.

Table S5A. Genes co-occupied by Fep1-5FLAG and Pho7-13MYC under iron-depleted conditions (RPKM ≥ 300; n = 264).

Table S5B. Genes co-occupied by Fep1-5FLAG and Pho7-13MYC under iron-replete conditions (RPKM ≥ 300; n = 484).

Table S5C. Genes co-occupied by Fep1-5FLAG and Pho7-13MYC under phosphate-replete conditions (RPKM ≥ 300; n = 32).

Table S5D. Genes co-occupied by Fep1-5FLAG and Pho7-13MYC under phosphate-depleted conditions (RPKM ≥ 300; n = 275).

Table S5E. Genes showing increased Pho7-13MYC binding under iron-replete versus iron-depleted conditions (FC ≥ 1.5; n = 226).

Table S5F. Genes showing increased Fep1-5FLAG binding under iron-depleted versus iron-replete conditions (FC ≥ 1.5; n = 36).

Table S5G. Genes showing increased Pho7-13MYC binding under iron-depleted versus iron-replete conditions (FC ≥ 1.5; n = 32).

Table S6. Effect of iron availability on co-enrichment of Fep1 and Pho7.

Table S6A. Genes co-enriched for Fep1-5FLAG and Pho7-13MYC binding under iron-replete conditions (FC ≥ 1.5; n = 178).

Table S6B. Genes co-enriched for Fep1-5FLAG and Pho7-13MYC binding under iron-depleted conditions (FC ≥ 1.5; n = 25)
